# Methylation marks in blood DNA reveal breast cancer risk in patients fulfilling hereditary disease criteria

**DOI:** 10.1038/s41698-024-00611-z

**Published:** 2024-06-19

**Authors:** Miguel Ruiz-De La Cruz, Héctor Martínez-Gregorio, Clara Estela Díaz-Velásquez, Fernando Ambriz-Barrera, Norma Gabriela Resendiz-Flores, Rina Gitler-Weingarten, María Patricia Rojo-Castillo, Didier Pradda, Javier Oliver, Sandra Perdomo, Eva María Gómez-García, Aldo Hugo De La Cruz-Montoya, Luis Ignacio Terrazas, Gabriela Torres-Mejía, Fidel de la Cruz Hernández-Hernández, Felipe Vaca-Paniagua

**Affiliations:** 1 Laboratorio Nacional en Salud, Diagnóstico Molecular y Efecto Ambiental en Enfermedades Crónico-Degenerativas, Facultad de Estudios Superiores Iztacala, Tlalnepantla, 54090 Mexico; 2 Unidad de Biomedicina, Facultad de Estudios Superiores Iztacala, UNAM, Tlalnepantla, 54090 Mexico; 3 Centro de Investigación y de Estudios Avanzados IPN (CINVESTAV). Avenida Instituto Politécnico Nacional #2508, Colonia San Pedro Zacatenco, Delegación Gustavo A. Madero, Departamento de Infectómica y Patogénesis Molecular, Ciudad de México, Mexico; 4 Fundación Alma I.A.P., 11560 Ciudad de México, México; 5 Instituto Nacional de Rehabilitación, Chorrillos, Peru; 6 Institute for Health Equity Research, Department of Health Science and Policy and Department of Environmental Medicine and Public Health at the Icahn School of Medicine at Mount Sinai, New York, NY USA; 7 Medical Oncology Service, Hospitales Universitarios Regional y Virgen de la Victoria, Institute of Biomedical Research in Malaga, CIMES, University of Málaga, 29010 Málaga, Spain; 8 Genomic Epidemiology Branch, International Agency for Research on Cancer (IARC/WHO), 150 Cours Albert Thomas, 69372 Lyon, France; 9 Centro de Tratamiento de Cancer Metepec, Toluca, 50180 Mexico; 10 Instituto Nacional de Salud Pública, Cuernavaca, 62100 Mexico

**Keywords:** Cancer epigenetics, Cancer genomics

## Abstract

Less than 15–20% of patients who meet the criteria for hereditary breast and ovarian cancer (HBOC) carry pathogenic coding genetic mutations, implying that other molecular mechanisms may contribute to the increased risk of this condition. DNA methylation in peripheral blood has been suggested as a potential epigenetic marker for the risk of breast cancer (BC). We aimed to discover methylation marks in peripheral blood associated with BC in 231 pre-treatment BC patients meeting HBOC criteria, testing negative for coding pathogenic variants, and 156 healthy controls, through methylation analysis by targeted bisulfite sequencing on 18 tumor suppressor gene promoters (330 CpG sites). We found i) hypermethylation in *EPCAM* (17 CpG sites; *p* = 0.017) and *RAD51C* (27 CpG sites; *p* = 0.048); ii) hypermethylation in 36 CpG-specific sites (FDR *q* < 0.05) in the BC patients; iii) four specific CpG sites were associated with a higher risk of BC (FDR *q* < 0.01, Bonferroni *p* < 0.001): cg89786999-*FANCI* (OR = 1.65; 95% CI:1.2–2.2), cg23652916-*PALB2* (OR = 2.83; 95% CI:1.7–4.7), cg47630224-*MSH2* (OR = 4.17; 95% CI:2.1–8.5), and cg47596828-*EPCAM* (OR = 1.84; 95% CI:1.5–2.3). Validation of cg47630224-*MSH2* methylation in one Australian cohort showed an association with 3-fold increased BC risk (AUC: 0.929; 95% CI: 0.904–0.955). Our findings suggest that four DNA methylation CpG sites may be associated with a higher risk of BC, potentially serving as biomarkers in patients without detectable coding mutations.

## Introduction

Breast cancer (BC, OMIM#114480) is a highly heterogeneous and multifactorial disease that is the primary cause of cancer-related mortality among women worldwide^1^. Hereditary breast and ovarian cancer syndrome (HBOC) is the most prevalent hereditary form of BC, accounting for up to 10-15% of all cases^2^, frequently associated with pathogenic variants in *BRCA1/2* genes^3,4^. Pathogenic variants in other genes, including *BRIP1, CHEK2, ATM*, and *PALB2* have been identified in less than 5% of cases^5^, demonstrating a significant locus heterogeneity.

The contribution of epigenetic mechanisms in BC development, and their ability to modulate gene expression independently of coding region mutations have been extensively documented^6^. Among epigenetic marks, DNA methylation has been widely studied in cancer tissues, observed in more than 70% of tumor suppressor gene (TSG) promoters in CpG contexts (5-methylcytosine) within regions known as CpG islands^7^. Aberrant methylation patterns in promoter regions, known as epimutations^8^, lead to the transcriptional silencing of TSGs or the activation of oncogenes, resulting in loss or gain of gene function, respectively. Epimutations causing diseases are referred to as constitutional epimutations. Epimutations can be categorized based on their origin as somatic or germline, and mechanistically as primary or secondary. Primary epimutations arise from stochastic or unknown causes, while secondary epimutations result from *cis* or *trans*-acting mutations. Both primary and secondary epimutations can occur in multiple embryonic and adult tissues, leading to epigenetic mosaicism^9^.

At the molecular level, altered DNA methylation marks in the BC tissue occur in early stages of the carcinogenic development^10^. In addition, DNA methylation alterations at genomic level have been observed in peripheral blood of individuals meeting HBOC criteria, revealing specific CpG sites associated to BC risk^11,12^. In the last decade, genome-wide DNA methylation in peripheral blood has been investigated in cases with familial BC in TSGs like *BRCA1, BRCA2*, *CHEK2, ATM, TP53, CDH1* and *MLH1*^13^. Increased global methylation has been associated with reduced risk of BC, while elevated DNA methylation within functional promoters with increased risk^14,15^. Moreover, aberrant germline hypermethylation of the *KIF1*^16^, *NDRG1*^17^, *ATM*^18^, and *PALB2*^19^ promoters has been proposed as a biomarker for BC and HBOC risk in some populations. However, current studies have focused on limited number of genes and HBOC patients, and have not analyzed methylation in patients without genetic pathogenic variants evaluated in large gene panels.

Previously, we reported that in a cohort of 300 BC patients with HBOC criteria, 15% had coding pathogenic variants, 11% had variants of uncertain significance (VUS), and 74% were negative through the analysis of 143 cancer susceptibility genes^20^. Therefore, we hypothesized that alternative mechanisms might increase the risk for BC in patients that fulfill HBOC criteria in whom no genetic coding alterations were identified. Hence, in this study, we aim to identify CpG sites exhibiting aberrant methylation patterns in peripheral blood and explore their association with BC risk. We employed a state-of-the-art analysis of the methylation status in the promoter regions of 18 well-known TSGs in 231 BC patients with HBOC criteria and negative for pathogenic variants or VUS, and compared them with 156 healthy women population controls.

## Results

### Clinical and epidemiological characteristics of the study population

**Fig. 1:**
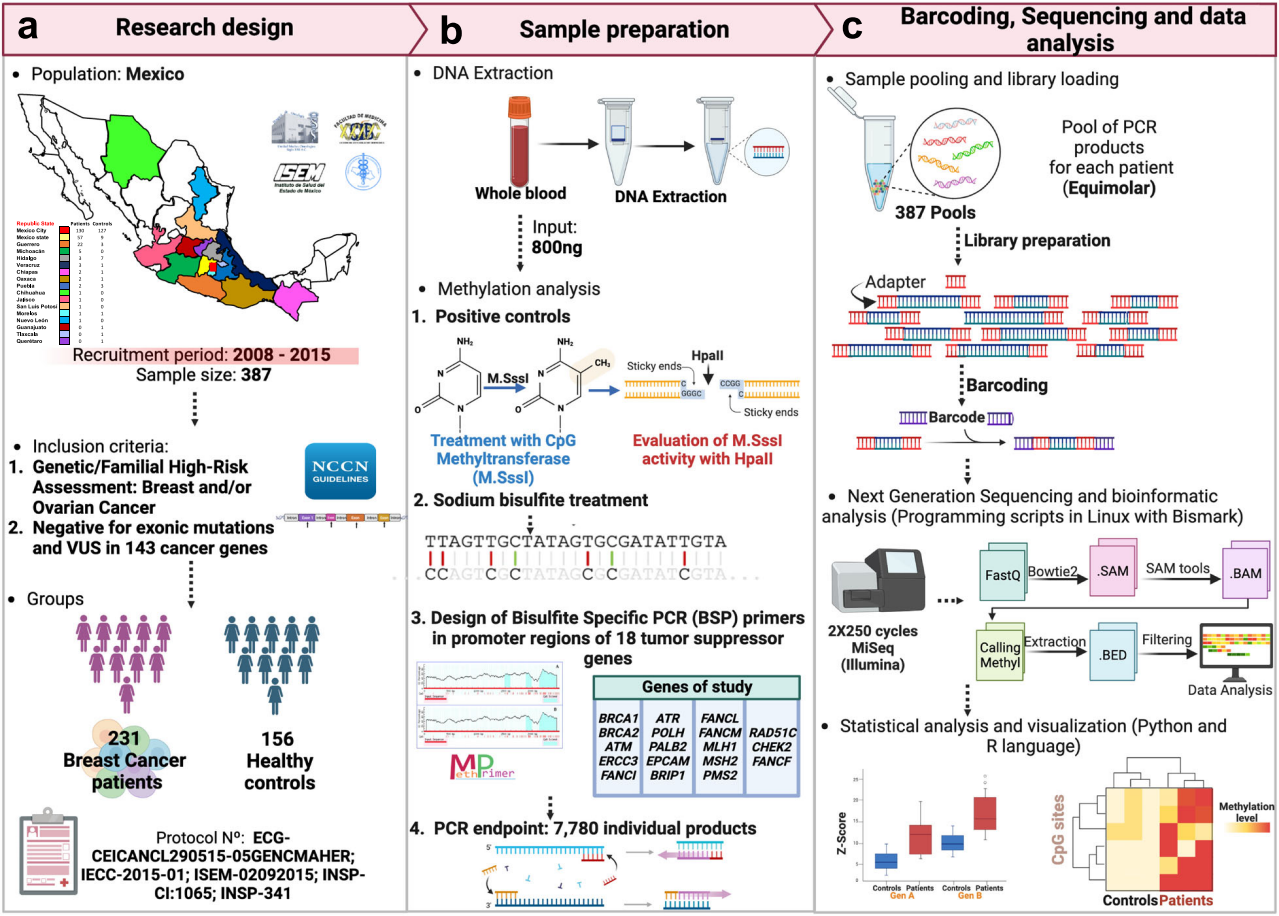
Experimental design. The recruitment and inclusion criteria for patient selection are shown in **a**. Sample preparation, bisulfite treatment, the targeted promoters, and total amplicons analyzed are presented in **b**. The DNA library preparation workflow, barcoding, automated bioinformatics analysis, and biostatistical analyzes are represented in **c**. Created with BioRender.com.

We selected 231 Mexican high-risk patients with BC, negative for coding pathogenic variants in 143 cancer susceptibility genes from a previous study conducted by our group^20^, and 156 healthy population controls (Fig. 1). There were significant differences in the average age (41.8 ± 7.4 vs 39.1 ± 4.8; *p* = 0.002, Wilcoxon rank-sum test), menarche (12.7 ± 1.5 and 12.4 ± 1.6; *p* = 0.008, Wilcoxon rank-sum test), menopause (30.7% vs 4.5%; *p* < 0.0001, chi-square test) and family history of cancer (83.5% vs 21.8%; *p* < 0.0001, chi-square test) (Table 1).

**Table 1:** Epidemiological and clinical features of the participants

Epidemiological features		Cases		Controls			*p*-value
		*N* = 231	100%		*N* = 156	100%	
Age		Mean ± S.D.	*n* (%)		Mean ± S.D.	*n* (%)	
	<40 years	34.5 ± 3.6	76 (32.9)		34.4 ± 3.9	66 (42)	0.746^a^
	40–50 years	43.4 ± 2.5	123 (53.2)		42.4 ± 1.7	91 (58)	0.002^a^
	>50 years	58.6 ± 5.2	20 (8.7)		N/A	0 (0)	N/A
	Unknown	N/A	12 (5.2)		N/A	0 (0)	N/A
BMI	<18.5 underweight	17.5 ± 1.2	2 (0.9)		N/A	0 (0)	N/A
	18.5 < 25 normal weight	23 ± 1.3	73 (31.6)		22.4 ± 1.7	25 (16)	0.159^a^
	25 < 30 overweight	27.5 ± 1.5	91 (39.4)		27.7 ± 1.5	64 (41)	0.610^b^
	30 < 40 obesity	32.8 ± 2.4	49 (21.2)		33.3 ± 2.4	55 (35.3)	0.260^a^
	>40 extreme obesity	46.7 ± 1.0	2 (0.9)		47.1 ± 5.9	12 (7.7)	N/A
	Unknown	N/A	14 (6.1)		N/A	0 (0)	N/A
Menarche	<12 years. Early	10.5 ± 0.8	42 (18.2)		10.4 ± 0.9	37 (24.2)	0.714^a^
	12 < 14 years. Normal	12.9 ± 0.8	148 (64.1)		12.6 ± 0.7	102 (65)	0.008^a^
	>14 years. Late	15.3 ± 0.8	26 (11.3)		15.5 ± 0.9	17 (10.8)	0.283^a^
	Unknown	N/A	15 (6.5)		N/A	0 (0)	N/A
Menopause		n	%		n	%	
	Yes	71	30.7		7	4.5	1.03E−10^c^
	No	147	63.6		149	95.5	
	Unknown	13	5.6		0	0.0	N/A
Family history of cancer^d^	Yes	193	83.5		34	21.8	2.2E−16^c^
	No	33	14.3		122	78.2	
	Unknown	5	2.2		0	0.0	N/A

Clinical features		Cases	
		*N* = 231	100%
		*n*	%
Histopathological subtype	DCIS	26	11.3
	LCIS	1	0.4
	IDC	160	69.3
	ILC	7	3
	MC	1	0.4
	Unknown	36	15.6
Clinical stage	I	25	10.8
	II	103	44.6
	III	61	26.4
	IV	6	2.6
	Unknown	36	15.6
Estrogen receptor	Negative	48	20.8
	Positive	105	45.5
(ER staining)	Unknown	78	33.8
Progesterone receptor	Negative	46	19.9
	Positive	107	46.3
(PgR staining)	Unknown	78	33.8
HER2 staining	Negative	122	52.8
	Positive	29	12.6
	Unknown	80	34.6
Molecular subtype	Luminal A	91	39
	Luminal B	23	10
	HER2+	7	3
	Triple Negative	32	13.9
	Unknown	78	33.8
Mutational status^e^	Not Mutated	231	100
	Mutated	0	0

*BMI* Body Mass Index, *DCIS* Ductal Carcinoma in situ, *LCIS* lobular carcinoma in situ, *IDC* invasive ductal carcinoma, *ILC* invasive lobular carcinoma, *MC* medullary carcinoma, *ER* estrogen receptor, *PR* progesterone receptor, *N/A* Data not available or not applicable.

^a^Wilcoxon rank-sum test.

^b^t-test.

^c^Chi-square test.

^d^The 231 cancer patients have a family history of breast, ovarian and cancers associated with HBOC, based on the NCCN criteria. The 156 healthy controls do not have family history of BC but may have family history of other types of cancer.

^e^The mutational status according to the presence of a pathogenic genetic variant in any of the 143 genes analyzed^20^.

The most common clinicopathological characteristics of the patients were invasive ductal carcinoma (69.3%), clinical stage II (44.6%), estrogen receptor positivity (45.5%), progesterone receptor positivity (46.3%), HER2 receptor negativity (52.8%), and Luminal A molecular subtype (39%) (Table 1).

### High-throughput DNA methylation sequencing reveals aberrant methylation in tumor suppressor genes in high-risk breast cancer patients

**Fig. 2:**
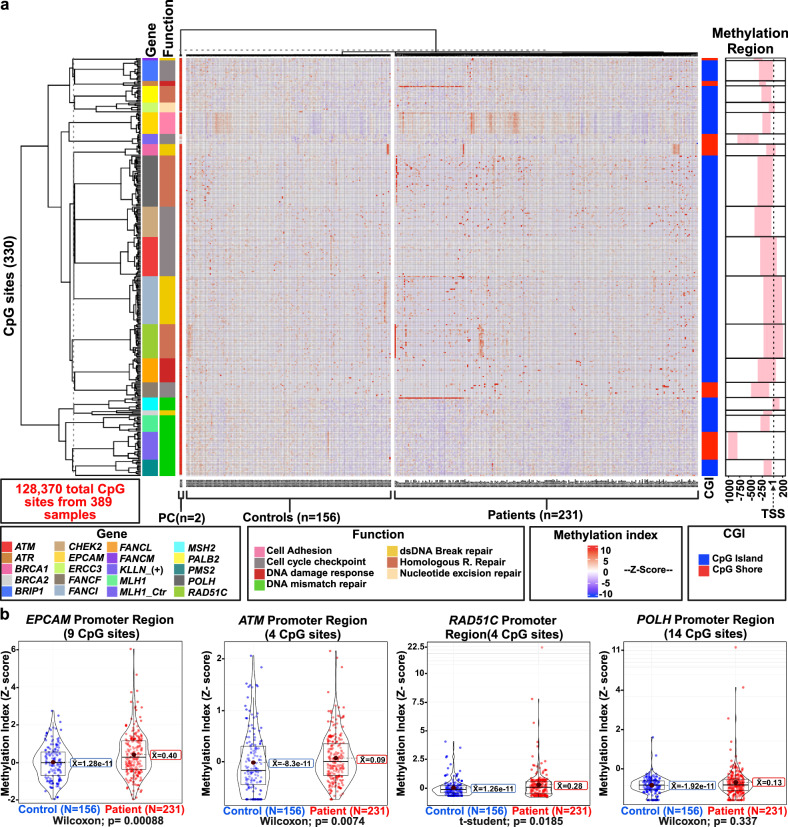
Identification of hypermethylated CpG sites in promoter regions of tumor suppressor genes in breast cancer. **a** Site-specific methylation status in the promoter regions of the analyzed TSGs. The supervised hierarchical clustering shows 128,370 CpG sites from 330 individual CpG sites in tested genes (Y-axis) relative to case-control samples (X-axis). Gene function and CpG island distribution are illustrated in the left and right panels, respectively. Methylation values were normalized with the Z-score. **b** Differential levels of hypermethylated CpG sites in *EPCAM*, *ATM*, *RAD51C*, and *POLH*. TSS transcriptional start site, CGI CpG island relative site, PC positive controls, TSGs tumor suppressor genes.

We evaluated the methylation levels of the promoter region of 18 TSGs in the 231 high-risk BC patients, and 156 healthy controls (Supplementary Tables 1, 2, Fig. 2a) by bisulfite sequencing PCR-NGS. In addition, *MLH1* and *KLLN* were used as internal controls for bisulfite conversion and full methylation, respectively.

We mapped 330 CpG sites in the 18 genes (128,370 CpG total sites in all samples). Systematic inspection with the Revelio software showed that none of the evaluated CpG sites had SNPs (Supplementary Figs. 2–6; see Materials and methods). In addition, no SNPs in the target region were detected using the dbSNP track in the UCSC Genome Browser. Then, we evaluated the average methylation values across all CpG sites (Supplementary Table 3), and found higher methylation in *EPCAM*, encompassing 17 CpG sites (Z-score of 0.326 vs −3.74E−16, *p* = 0.017, Wilcoxon rank-sum test), and *RAD51C* (27 CpG sites; Z-score of 0.132 vs to −1.17E−16, *p* = 0.048).

A following analysis of specific CpG sites identified 36 hypermethylated marks (FDR *q* < 0.05) in 9 genes (Supplementary Table 4). Statistical analysis was done on the genes with more than two hypermethylated CpG sites, which included *ATM* (4 CpG sites; Z-score 0.07 vs 1.34E−17, *p* = 0.0074), *RAD51C* (4 CpG sites; Z-score 0.03 vs 2.38E−18, p = 0.018), and *EPCAM* (9 CpG sites; Z-score 0.42 vs 1.37E−17, *p* = 0.00088) (Fig. 2b). In addition, locus-wide hypermethylation in the *RAD51C* (mean methylation: 23.5%; 27 CpGs), *BRCA1* (mean methylation: 9.6%; 9 CpGs), and *POLH* (mean methylation: 9.1%; 33 CpGs) was detected in three patients.

### Breast cancer patients showed enriched hypermethylation in four specific CpG sites

**Fig. 3:**
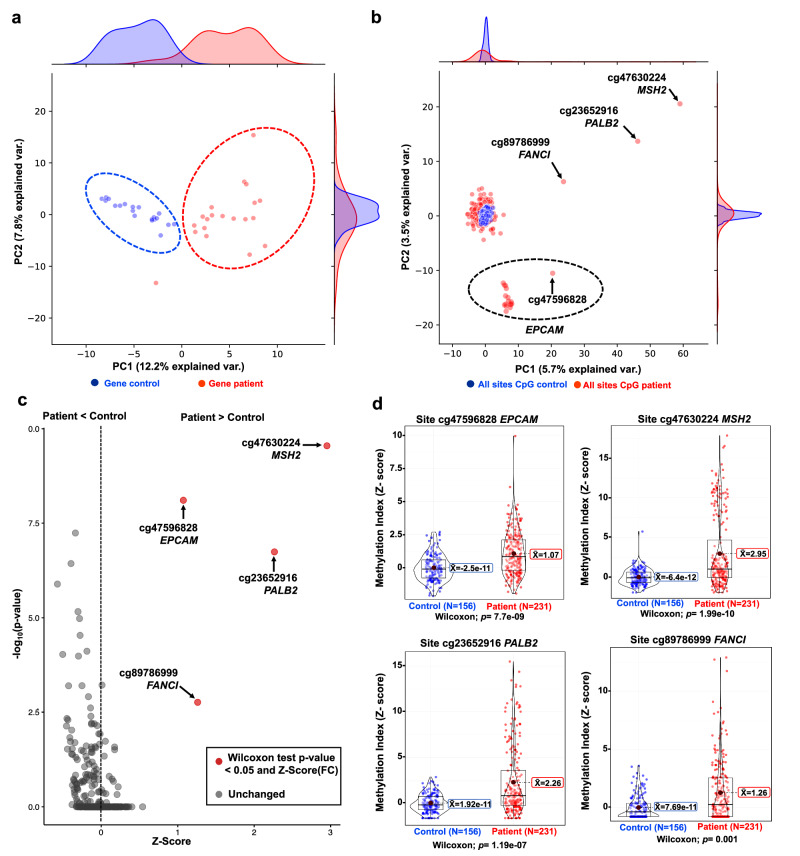
Four aberrant DNA methylation marks are associated with breast cancer. **a** Principal component analysis of the mean Z-score for each promoter. **b** PCA of the individual 330 sites. The cg8978699-*FANCI*, cg23652916-*PALB2*, cg47630224-*MSH2*, and cg47596828-*EPCAM* had the highest variation (arrows). All *EPCAM* sites formed an independent group. **c** Differential hypermethylation levels of the individual CpG sites. Red dots represent significant sites. **d** Differential hypermethylation of the significant CpG sites as compared with controls. Red boxes indicate controls and blue boxes indicate cases. Sites with FDR *q* < 0.05 and Bonferroni strict correction *p* ≤ 0.001 were considered significant. Dotted lines indicate the k-means per group in **a** and **b**.

**Fig. 4:**
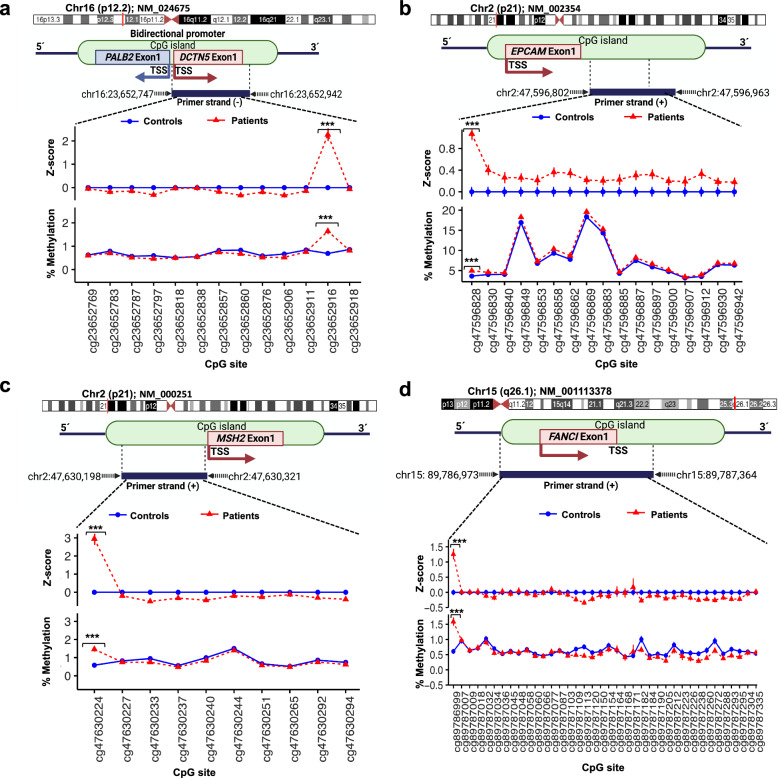
Comparison of site-specific germline methylation levels across the promoter region of *PALB2, EPCAM*, *MSH2*, and *FANCI* in patients vs controls. **a**–**d** The *loci* of the four genes are shown. The solid blue line indicates the average methylation values for DNA derived from blood controls. The dotted orange line indicates the average methylation values for patients. The sites cg47596828-*EPCAM*, cg47630224-*MSH2*, cg23652916-*PALB2* and cg89786999-*FANCI*, showed a statistically significant methylation level increase in patients. Schematics of the gene region denote CGI CpG island, TSS transcriptional start site. Triple asterisk (***) represent *p* < 0.001.

To identify general methylation patterns in the global normalized methylation levels of all 18 TSGs, we conducted a PCA. Using the differentiated Z-scores, the methylation profile of the gene promoters for patients and controls was classified into two distinct clusters (Fig. 3a). At the individual CpG site level, we identified hypermethylation in patients compared to healthy controls in four sites: i) cg47630224-*MSH2* (Z-score 2.95 vs −6.41E−12, *p* = 1.99E−10; *q*-value = 2.23E−11), ii) cg23652916-*PALB2* (Z-score 2.27 vs 1.92E−11, *p* = 1.19E−07; *q*-value = 3.34E−09), iii) cg89786999-*FANCI* (Z-score 1.3 vs 7.69E−11, *p* = 001; *q*-value = 1.10E−05), and iv) cg47596828-*EPCAM* (Z-score 1.1 vs −2.56E−11, *p* = 7.74E−09; *q*-value = 4.33E−10), (Fig. 3b–d, Fig. 4, Supplementary Table 5). Notably, all CpG sites in the *EPCAM* gene exhibited higher methylation levels compared to controls (Fig. 3b).

**Fig. 5:**
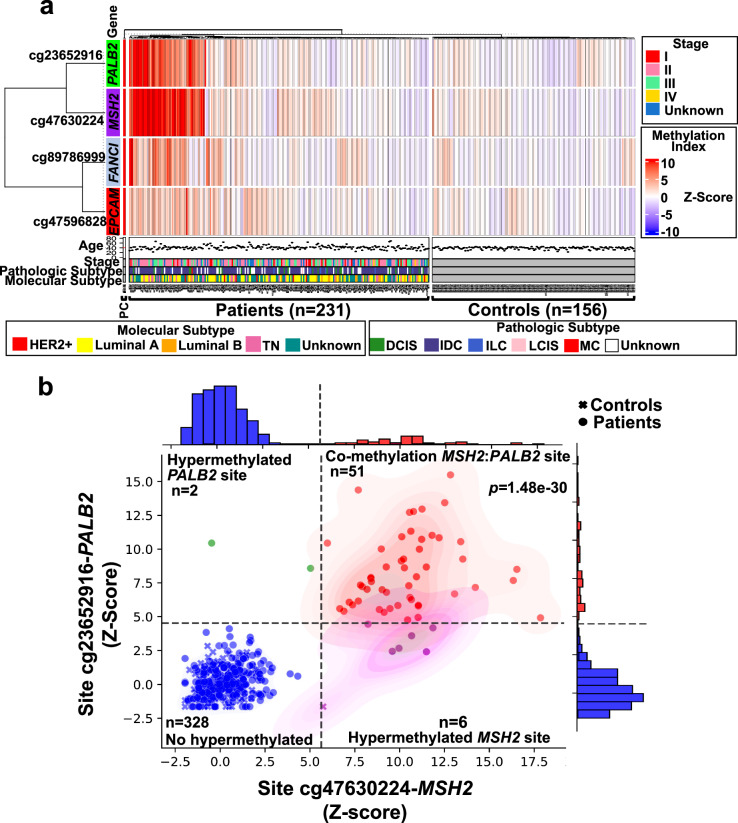
Co-methylation of the cg23652916-*PALB2* and cg47630224-*MSH2* sites. **a** Supervised hierarchical clustering heatmap shows a subclade with *MSH*-*PALB2* co-methylation in 51 patients. Y-axis: CpG sites. X-axis: controls and patients. The lower strips show the age, histopathological and molecular subtype of each patient in relation to each hypermethylated site. DCIS ductal carcinoma in situ, LCIS lobular carcinoma in situ, IDC invasive ductal carcinoma, ILC invasive lobular carcinoma, MC medullary carcinoma, PC positive controls. **b** Z-score co-methylation levels of cg23652916-*PALB2* and cg47630224-*MSH2*. Dotted line: two-sided +/−1 STD of Z-score. *p* < 0.001 (Wilcoxon signed-rank test). Controls are depicted as crosses and patients as dots.

The distribution of the methylation levels at the four significant hypermethylated sites was assessed by a supervised hierarchical clustering analysis. Two clades resulted, one with clear hypermethylation and one with lesser levels of methylation (Fig. 5a). Within the clade of high methylation, there was a subgroup of 22% of patients (51/231; cut-off two-sided +/−1 STD of Z-score [4.6, *PALB2*; 5.6 *MSH2*]) with co-methylation of the cg23652916-*PALB2* and cg47630224-*MSH2* sites (Fig. 5b). When exploring the molecular characteristics of this subset of patients, we found that 22% (13/58) present a triple-negative subtype. The clinical stages of these patients were stage I 17% (10/58), stage II 29.5% (17/58), stage III 24% (14/58), 29.5% (17/58) was not reported (Fig. 5a).

### Four specific hypermethylated CpG sites as potential biomarkers of breast cancer risk

**Fig. 6:**
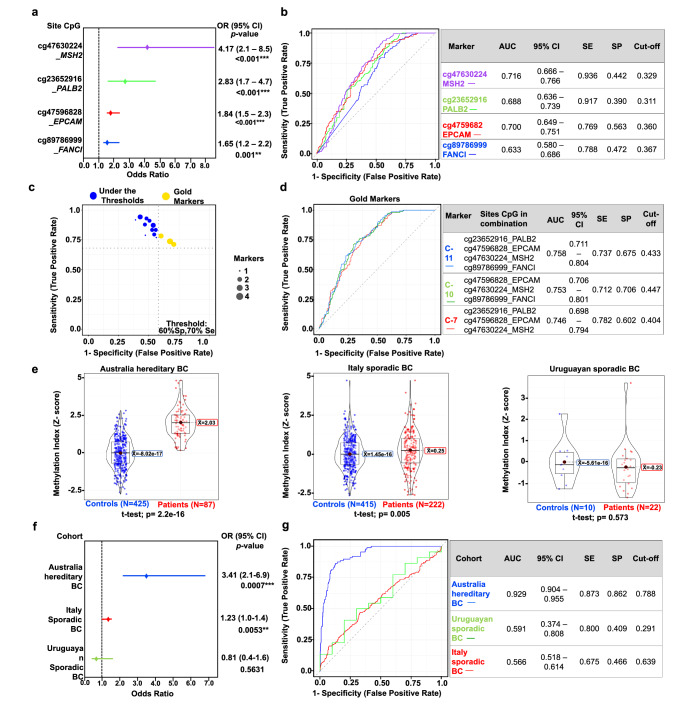
Biomarker capacity of the four CpG sites and association between the cg47630224-*MSH2* and breast cancer risk in independent validation cohorts. **a** Breast cancer risk of the four hypermethylated CpG sites. **b** ROC performance of the four CpG sites. **c** Combinatorial analysis of sensitivity and specificity for 11 CpG site combinations. Yellow bubbles represent the best marker combinations (Se >70%, Sp >60%), referred to as “gold markers”. **d** Biomarker capacity of gold marker combinations 7, 10, and 11. **e** Differential methylation analysis of cg47630224-*MSH2* in three international cohorts. **f** Association of cg47630224-*MSH2* site with BC risk in three cohorts. **g** Biomarker capacity of cg47630224-*MSH2* site in all cohorts: Australia (blue), Uruguay (green), Italy (red). BC breast cancer, OR Odds Ratio, 95% CI 95% confidence intervals, AUC area under curve, Sp specificity, Se sensitivity. Double asterisk (**) for *p* < 0.01, triple asterisk (***) for *p* < 0.001.

Increased global methylation has been associated with a reduced risk for BC, while abnormal DNA hypermethylation within functional promoters to increased risk^14,15^. Therefore, we aimed to confirm the association between the four aberrant DNA methylation markers and BC risk through univariate and multivariate logistic regression analyses of binomial odds ratios (ORs), utilizing both raw methylation data and Z-scores (Supplementary Tables 6–11). The multivariate analysis was adjusted for age, age at menarche, BMI, family history of cancer, and depth of sequencing. DNA hypermethylation at the sites cg47596828-*EPCAM* (OR = 1.84 [1.46–2.32], 95% CI, *p* < 0.001), cg47630224-*MSH2* (OR = 4.17 [2.05–8.48], 95% CI, *p* < 0.001), cg23652916-*PALB2* (OR = 2.83 [1.71–4.66], 95% CI, *p* < 0.001), and cg89786999-*FANCI* (OR = 1.65 [1.24–2.20], 95% CI, *p* = 0.001) was associated with an increased risk of BC, using the raw methylation data (Fig. 6a).

Interquartile analysis revealed that higher incremental progressive quartiles were linked to a greater risk of BC for each CpG site (Supplementary Tables 8–11). To assess the potential of these CpG sites as biomarkers for BC, we performed a receiver operating characteristic (ROC) analysis. The sites cg47596828- *EPCAM* (AUC: 0.700, Cut-off: 0.360) and cg47630224-*MSH2* (AUC: 0.716, Cut-off: 0.329) showed the highest performance (Fig. 6b). Additionally, a combinatorial ROC analysis using all four aberrant methylation marks (11 combinations) showed three optimal combinations referred to as gold markers: combination 7, 10, and 11 (Fig. 6c, d, Supplementary Fig. 7).

### Validation of the potential CpG biomarkers in three independent cohorts

To validate the results of our analysis for significant CpG sites, we conducted a comprehensive search for studies on methylation analysis that targeted the same sites. Our search revealed that the Infinium HumanMethylation450 (HM450K) BeadChip-Illumina was the only platform with structured methylation data available for BC, covering over 480,000 CpG sites. However, the design of this technology only includes the cg47630224-*MSH2* (Illumina probe cg22269526), which was used to validate our findings. We initially screened 1786 articles related to BC using the NCBI-GEO (https://www.ncbi.nlm.nih.gov/gds) database and filtered them to 74 articles that included methylation profiles (case-control). We excluded studies that involved cell lines or chemotherapy treatment, resulting in three relevant cohorts: the Australian cohort GSE104942 (87 BC patients with HBOC criteria), the EPIC-Italy (HuGeF) cohort GSE51032 (222 sporadic patients), and the Uruguayan cohort GSE148663 (22 sporadic patients).

Higher methylation levels in the cg47630224-*MSH2* site were observed in the Australian hereditary BC cohort (mean Z-score patients = 1.971 vs −8.65E−8, *p* < 0.0001) and the Italian sporadic cases cohort (mean Z-score patients = 0.238 vs −0.016, *p* = 0.0057). Conversely, no significant differences were found in the Uruguayan sporadic cases cohort (Z-score patients = 0.529 vs −0.6518, *p* = 0.573) (Fig. 6e).

Furthermore, logistic regression analysis showed an association with BC risk in the Australian cohort (OR: 3.41, 95% CI: 2.1–6.9, p < 0.001) compared to the cohorts from Italy (OR: 1.23, 95% CI: 1–1.4, *p* < 0.01) and Uruguay (OR: 0.81, 95% CI: 0.4–1.6, *p* > 0.05) (Fig. 6f). In the ROC analysis, we observed an AUC of 0.892 (Sensitivity: 0.900, Specificity: 0.885) in the Australian cohort (Fig. 6g).

## Discussion

Prior studies have shown that around 70% of patients who meet NCCN criteria for HBOC lack pathogenic variants in cancer susceptibility genes^20,21^. This led us to hypothesize the existence of alternative molecular mechanisms on these patients. In this study, we aimed to identify epigenetic alterations in HBOC-eligible BC patients without pathogenic coding variants by DNA methylation analyses on 18 TSGs in peripheral blood from 231 Mexican patients and 156 healthy population controls.

Here, we detected hypermethylation in the promoter regions of *ATM*, *RAD51C*, and *EPCAM* genes, with implications for hereditary BC. In familial BC, *ATM* hypermethylation in peripheral blood DNA was associated with a 3-fold increased risk of bilateral BC (*p* = 0.0017)^13^, suggesting its potential as a novel marker for BC risk in individuals fulfilling HBOC criteria. Similar findings were observed for distinct *ATM* CpGs (OR: 1.89, *p* = 2 × 10^−4^)^18^. Constitutive *RAD51C* hypermethylation (>6%) was reported in HBOC patients^22^, but conflicting evidence exists^23^; therefore, further work is needed to determine its precise influence on BC risk. *EPCAM* mutations are known to cause methylation and transcriptional repression in the neighboring *MSH2* gene in hereditary colorectal cancer^24^, yet but there are no studies in BC patients with criteria for hereditary disease.

We discovered 36 specific hypermethylated marks associated with an increased risk of BC, including cg47630224-*MSH2*, cg23652916-*PALB2*, cg89786999-*FANCI*, and cg47596828- *EPCAM*. These CpG sites had not previously been linked to BC risk, which underscores the benefit of using open platforms such as NGS for evaluating genome-wide DNA methylation status in familial BC.

The link between blood DNA methylation in specific marks and BC patients with HBOC criteria has been examined. A previous work in Australian patients identified four heritable methylation CpG sites in *GREB1*, *PNKD*, *C7orf50* and *TMC3*, associated with BC risk in 210 individuals from 25 families^25^. A nested case-cohort analysis within the prospective Sister study revealed 250 individual CpG sites with differential methylation between BC cases and controls^11^. Only one CpG site in *ERCC1* was associated with an increased risk of BC in an independent cohort^26^. The potential implication of specific CpG site methylation in patients with hereditary cancer criteria was further supported by a comparative study between sporadic and hereditary BC patients lacking pathogenic variants from the same population. In this work, a CpG site in *BRCA1* showed significant hypermethylation in the hereditary cases^27^. Overall, these findings suggest that methylation at specific CpG sites could play a role in the development of the disease in affected women whose families meet criteria for HBOC.

Sporadic BC and peripheral blood DNA methylation have also been studied. An integrative genetic analysis of 122,977 sporadic BC patients and 105,974 controls revealed 38 CpGs potentially increasing BC risk by regulating 21 genes^28^. A Chinese study identified four CpG sites in imprinting genes *KCNQ1*, *KCNQ1OT1*, and *PHLDA2* associated with increased BC risk^29^. Conversely, a meta-analysis of four prospective cohorts found no evidence supporting individual CpG site methylation in blood as a sporadic BC risk factor^30^. Therefore, the link between methylation and sporadic BC remains unclear.

Among the four genes with altered hypermethylation, *MSH2* and *PALB2* showed the strongest association with BC. Interestingly, we observed *MSH2*-*PALB2* co-methylation in 51 of 57 patients with high *MSH2* methylation levels, suggesting a potential mutual association via an unknown biological mechanism (Fig. 5). Compelling evidence indicates *MSH2* promoter hypermethylation is induced by *cis EPCAM* gene rearrangements in Lynch syndrome patients^31^. Additionally, *PALB2* hypermethylation has been reported in 8% of sporadic breast and ovarian cancer patients^19^. A study on familial BC found significant tumor methylation in four CpG sites within the *MSH2* promoter region, including cg47630224-*MSH2* (Illumina probe cg06478094), associated with increased BC risk in our study^32^. Despite *MSH2* lack of current association with increased BC risk, *PALB2* is a well-established high-risk BC susceptibility gene. We hypothesize that the concurrent methylation at the specific sites of *PALB2* and *MSH2* in the germline of 22% patients might have a synergistic effect, possibly mediated by shared distant regulators, as other reports have proposed that co-methylation might be an indicator of functional associations between gene pairs in somatic BC^33–35^.

*Cis* mutations in TSGs are linked to locus-wide hypermethylation in colorectal cancer and other oncologic syndromes^9,24^. Interestingly, we observed this abnormal pattern in the promoter regions of *RAD51C* (23.5%), *BRCA1* (9.6%), and *POLH* (9.1%) in patients GT25, GT214, and GT202, respectively, suggesting neighboring mutations may induce hypermethylation. A single study investigated this mechanism in HBOC-criteria patients, finding a hypermethylated *BRCA1* allele (>35%) cosegregating with the variant c.-107A>T in the *BRCA1* 5′UTR^36^. However, validation of this variant failed in a large German cohort and in BC and ovarian tumors with *BRCA1* promoter hypermethylation, indicating suggesting low allelic frequency in German and Dutch patients^37,38^. In a follow-up study, we aim to explore the potential incidence and functional implications of these putative *cis* mutations.

To further support our findings, we assessed the aberrant methylation status of the cg47630224-*MSH2* site across three independent cohorts that used the Illumina Infinium 450 K methylation array platform. We confirmed the association of this CpG site with BC risk in the cohort of patients with HBOC criteria (Australia; OR: 3.41, *p* < 0.001), suggesting the potential biomarker capacity of our approach. However, more studies are needed to fully confirm the role of cg47630224-*MSH2* as a biomarker.

This study presents a thorough analysis, at single-base resolution, of high-risk gene promoters in a large case-control dataset, utilizing healthy population controls to minimize methylation level variability from non-genetic and environmental factors^39^. The limited availability of validation cohorts for the specific CpG sites we examined is a limitation of this study. Only one comparable report involving Australian patients was found for validation. Hence, we cannot fully exclude that this methylation marks are not associated with sporadic BC. Moreover, these findings should not yet be interpreted as indicative of an autosomal dominant trait, as the methylation patterns have not been assessed in family members. Additional limitations include the lack of assessment of dietary variables known to influence methylation changes, such as vitamin B12, folate, choline concentration^40^, tobacco use, and exposure to endocrine-disrupting substances^41^. Despite these limitations, our findings support previous studies and offer compelling evidence of site-specific methylation as a potential marker in BC patients meeting HBOC criteria, particularly in an underrepresented population in the epigenetic literature.

**Fig. 7:**
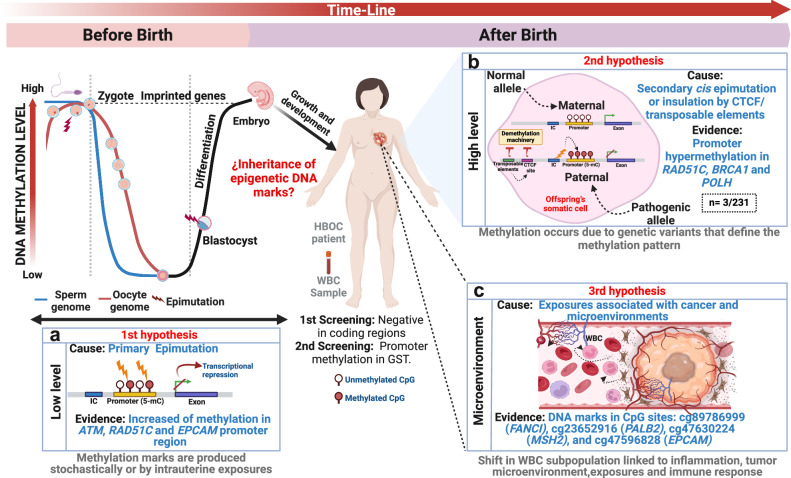
A model of aberrant methylation marks implicated in breast cancer risk. Somatic methylation is erased in the primordial germ cells of the sperm and oocyte and reprogramed during differentiation process of the blastocyst development and tissue differentiation. How do inherited epigenetic markers establish? **a** Hypothesis I: Aberrant methylation patterns may arise during embryonic development as a result of primary stochastic epimutations driven by intrauterine exposures. **b** Hypothesis II: Somatic-level reestablishment of methylation marks in the promoter region due to *cis* mutations in imprinting centers (IC) may lead to transcriptional silencing of the gene. Hypermethylation could also be maintained at somatic level by chromatin constitutive isolation due to the effect of neighboring insulator and transposable elements that contain CTCF sites. This might lead to the inability of the demethylating machinery to access these loci. **c** Hypothesis III: The tumor microenvironment and the immune response could potentially contribute to changes in leukocyte subpopulations by establishing methylation marks in the promoter of TSGs. Created with BioRender.com.

We summarize our findings in three hypotheses (Fig. 7). Increased low level methylation in *ATM*, *RAD51C*, and *EPCAM* promoter regions might arise from stochastic primary epimutations due to environmental factors during the embryonic development and adulthood (Fig. 7a)^42^. High level, locus-wide methylation in *RAD51C*, *BRCA1*, and *POLH* detected in three patients are product of non-coding genetic alterations in the neighboring regions, causing secondary *cis* epimutations (Fig. 7b)^9,24,36^. The aberrant methylation in specific CpG sites in *MSH2, PALB2, FANCI*, and *EPCAM* could result from changes in the DNA of peripheral blood leukocyte subpopulations, influenced by the tumor microenvironment, and the immune response at systemic level (Fig. 7c)^43^.

In conclusion, this study provides evidence regarding the association between germline methylation of cancer susceptibility genes in BC patients with criteria for HBOC without detectable coding pathogenic variants. We identified four novel potential epigenetic markers associated with a higher risk of BC, which were validated in an independent cohort. Overall, this work contributes to improving our understanding of the epigenetic landscape of high-risk BC patients, which could be alternative mechanisms of etiopathology. Further investigation into epigenetic signatures holds the potential to enhance risk assessment and facilitate personalized approaches in BC management.

## Methods

### Study population

From the Latin American Study of Hereditary Breast and Ovarian Cancer (LACAM) cohort, we selected 231 Mexican patients with BC that fulfill the *National Comprehensive Cancer Network* (NCCN) criteria for HBOC in 143 genes previously reported^20^. The inclusion criteria were: i) negative for pathogenic variants, ii) negative for VUS in clinically relevant genes, iii) with availability of more than 800 ng genomic DNA (gDNA), iv) sample obtained prior to chemotherapy treatment. We term these patients as high-risk BC patients. In addition, 156 healthy controls without a family history of BC were selected, including only those with family histories of other types of cancer. All participants signed an informed written consent for the use of their biological samples for research purposes. The research was conducted according to the Declaration of Helsinki and approved by the Ethics Committee of four health institutes in Mexico (Protocols: ECG-CEICANCL290515-05GENCMAHER, IECC-2015-01, ISEM-02092015, INSP-CI:1065, and INSP-341) (Fig. 1a).

### gDNA extraction from peripheral blood

gDNA was extracted from peripheral blood using the DNeasy Blood & Tissues kit (Qiagen, Hilden, Germany). The integrity of the gDNA was evaluated by electrophoresis in a 0.8% agarose gel and the purity in an EPOCH BIOTEK spectrophotometer. Quantification was performed using a Quantus Fluorometer with the dsDNA Quantifluor kit (Promega, Madison, USA) (Fig. 1b).

### Positive methylation controls

Two positive methylation controls were included: i) METC1-POS: a commercial Human HCT116 DKO Methylated DNA (Zymo Research, Irvine, CA, USA); and ii) METC2-POS gDNA, both treated in vitro with the enzyme DNA methyltransferase M. *Sss*I. The methylation percentages in the positive controls were 98-100% meaning complete methylation. The methylation status in positive controls was validated by methylation-sensitive restriction enzyme assay using *Hpa*II (New England Biolabs, UK). Methylated sites in CpG context block *Hpa*II activity. The detailed procedures are described in Supplementary Fig. 1.

### Methylation assay by bisulfite conversion

800 ng of gDNA from patients and controls were treated with Sodium Bisulfite using the EZ DNA Methylation-Gold kit (Zymo, California USA). The bisulfite-converted DNA was quantified with the ssDNA Quantifluor kit (Promega, Madison USA) and nanophotometer spectrophotometry (Implen), and stored at −20 °C for targeted bisulfite sequencing (Fig. 1b).

### Primer design for bisulfite sequencing PCR

We designed primers for the promoter regions of 18 TSGs: *ATM*, *ATR*, *BRCA1*, *BRCA2*, *BRIP1*, *CHECK2*, *EPCAM* (first intron CpG island), *ERCC3*, *FANCF*, *FANCI*, *FANCL*, *FANCM*, *MLH1*, *MSH2*, *PALB2*, *PMS2*, *POLH*, and *RAD51C* using the MethPrimer 2.0 online software. See supplementary methods and Supplementary Table 1 for the primer design criteria.

### Endpoint PCR amplification

We performed a total of 7780 endpoint PCRs to amplify the target regions within the promoters of the 18 TSGs (one amplicon per promoter). GoTaq Polymerase Master Mix® (Promega, Madison USA) was used for the amplification. Each reaction was performed in 25 μL as follows: 12.5 μL of GoTaq 2X Mix enzyme, primer forward and reverse were added to 200 nM, 20 ng of DNA converted with sodium bisulfite template and adjust to 25 μL total with RNAse-free water. A no-template reaction was used as a negative control. *MLH1* promoter region was used a positive control.

The thermal cycling conditions used for the PCR were: one initial denaturation cycle at 95 °C for 3 min; 40 cycles of denaturation at 95 °C for 30 s, alignment at the primer specific Tm for 30 s, extension at 72 °C for 30 s; a final extension step at 72 °C for 5 min, and the reaction was kept at 4 °C. Subsequently, the amplified products were resolved on a 0.8% agarose gel (Supplementary Table 1).

### Pooling, library preparation and next generation sequencing

The individual PCR products were equalized to 6.36 nM, and pooled by sample. We obtained 389 equimolar equalized PCR pools (231 patients, 156 controls, and 2 positive methylation controls) with 20 genes (18 study genes and 2 internal control genes). Each pool was purified with AMPure XP Beads (1.8X) and a total of 70 ng of DNA was used for the preparation of DNA libraries using the NEBNext Ultra™ II DNA Library Prep Kit for Illumina. The generation of high-quality DNA libraries was confirmed by Bioanalyzer 2100 High Sensitivity DNA ChiP analysis, (Agilent, California USA). The samples were paired-end sequenced (2 × 250) with a theoretical deep coverage of 10,000X, in a MiSeq instrument (Fig. 1c).

### Bioinformatics and quantitative methylation

DNA methylation was detected by an automated program. The pipeline performed the mapping of the CpG sites and filtering of specific-site methylation data with as follows: i) raw reads were assessed with MultiQC v1.13 and processed with Trim galore v0.6.6 keeping bases with Phred quality score >30; ii) mapping and methylation calling were done with Bismark v0.22.3^44^, using GRCh37/hg19 genome reference; and iii) regions of interest were kept for downstream analysis. The methylation values were expressed as percentage (0–100%) and normalized using the control samples, applying Z-score for each specific CpG site according to the following equation:

$${Z}_{{ij}}=\frac{{x}_{{ij}}-\bar{{x}_{j}}}{{S}_{j}}$$

where ${Z}_{{ij}}$ is the Z-score value of each CpG of each promoter (j) for each sample patient (i); ${x}_{{ij}}$: methylation of each CpG of the promoter evaluated (j) for each sample patient (i); $\bar{{x}_{j}}:$ mean methylation of each CpG of the promoter evaluated (j) from the control samples; and ${{S}}_{j}$: standard deviation of methylation control samples for each CpG of the promoter evaluated (j). This normalization approach has been applied in other reports to evaluate the methylation status of specific CpG sites in case-control studies^45–47^. A Z-score above zero was considered higher than the methylation control mean.

The detection of single nucleotide polymorphisms (SNPs) from bisulfite sequencing alignments was done with the Revelio software^48^ (Supplementary Figs. 3–7). SNPs were also evaluated with the dbSNP track in the UCSC Genome Browser.

### Statistical analysis

We conducted the Wilcoxon test with Bonferroni correction on the p*-*values of all CpG sites to control the Type I error rate in multiple comparisons. This analysis was carried out using scipy v1.10.1 and statsmodels v0.14. Differences with a *p*-value of less than 0.05 were considered statistically significant. Additionally, to correct for multiple testing, we set the false discovery rate (FDR) threshold at *q* < 0.05, employing the qvalue v2.32.0 library.

We performed a supervised hierarchical analysis with Pearson correlation and principal component analysis (PCA), stratified into patient and control groups, using ComplexHeatmap v.2.13.1 and scikit-learn v1.1.2 libraries, respectively. The relation between Z-score and the p-value obtained from the Wilcoxon rank-sum test were depicted in a volcano plot to visualize individual site methylation fold-change differences. Univariate and multivariate logistic regression model Odds Ratio (OR) analyses were conducted to analyze the association between the peripheral blood methylation of each CpG site and the risk of BC and were calculated using OddsPlotty v1.0.2 and Stata V18.0. The results are represented as odds ratios (ORs) and 95% confidence intervals (95% CIs). Finally, we made a classifier with the significant sites individually and combined, and specificity and sensitivity were calculated with pROC v1.18.0 and CombiROC v0.2.3, respectively.

### Validation in three independent cohorts

We used three datasets from NCBI-GEO, the cohorts EPIC-Italy (HuGeF) GSE51032 (222 sporadic patients), GSE148663 from Uruguay (22 sporadic patients), and the Australian cohort GSE104942 (87 BC patients with HBOC criteria), analyzed with Infinium HumanMethylation450 (HM450K) BeadChip-Illumina arrays. Samples displaying low yield of detection (*p* > 0.05) in the Infinium cg22269526 probe were excluded from the analysis across all three cohorts. The obtained β-values derived from the HM450K platform were expressed as percentages, with 0 indicating 0% methylation and 1 indicating 100% methylation. These values were subsequently normalized using Z-score. The univariate logistic regression model Odds Ratio (OR) and ROC curves were calculated as described above.

### Supplementary information

Supplementary Information

## Data Availability

The data presented in the study are deposited in the Sequence Read Archive repository, accession number PRJNA987643 and PRJNA987641.

## Code availability

The codes used in this study are deposited in https://github.com/UBIMED-Lab13/MethylationDetection.git.
