# Supplementary material for: Methylation marks in blood DNA reveal breast cancer risk in patients fulfilling hereditary disease criteria
Source: NPJ Precis Oncol. 2024 Jun 19;8:136. doi: 10.1038/s41698-024-00611-z (PMC11187128; doi:10.1038/s41698-024-00611-z)
Supplement: Supplementary file 1 — Supplementary Information [file 41698_2024_611_MOESM1_ESM.pdf]

Supplementary material

The primer design was realized by thoroughly considering the following specific criteria: i) exclusion of CpG sites within the primer sequence to ensure unbiased amplification of methylated or unmethylated DNA, and ii) when CpG island prediction was not used for primer selection (default setting), PCR products were required to cover a minimum number of specific CpG sites (range: 2-40 CpG sites). Primer design can be found in **Table S1**. The *MLH1* oligonucleotides were utilized as an internal positive control to validate the sodium bisulfite conversion, following the protocol of the EZ DNA Methylation-Gold kit.

Table S1. Sequences of primers and products of targeted methylation sequencing

				Product information		
**Gene***	**mRNA**	**T[a] ºC**	**Primer sequence (Bisulfite Sequencing PCR)**	**Genomic location**	**Product size(bp)**	**Strand**
EPCAM	NM_002354	54.6	F: GTATAATAGGGAGGGGATTAAGAGGT<br>R: AAACCATTTCCCTACCAAAA	chr2:47596802 – 47596963	162	+
BRIP1	NM_032043	53	F: TAAAGAATTTGGTAAAGTTTAAAGA<br>R: AACCAAAAATCAACCAATCC	chr17:59940949 – 59941237	289	-
ATR	NM_001184	53	F: TTTTTAGAAAGAGGGATAAGAG<br>R: CACAAATATAAAAACTATAACAATCCAA	chr3:142297947 – 142298077	129	-
ATM	NM_000051	53	F: ATAGTAGGAATTATAATAAGGAATAAGAT<br>R: CCATATCCACCAATAACCAA	chr11:108093274 – 108093619	346	+
ERCC3	NM_001303418	53	F: TTTGTGTGGATAATAGGGAGT<br>R: CATAACAACTACAACAACAAAAA	chr2:128051655 – 128051766	112	-
RAD51C	NM_002876	54.6	F: GGAGAATTTATTGGGTTTGG<br>R: CCTCTAAAAATTCCTCAACAATCTAAA	chr17:56769721 – 56770125	405	+
FANCF	NM_022725	53	F: AAGGATAATGTGAAGGTTTAGAAT<br>R: CCTTAACTCTAAACAACTTAAAAACCCTA	chr11:22647502 – 22647882	381	-
FANCL	NM_018062	52.3	F: GGATAATAGTAAAGAAGATAGAGTGTTAT<br>R: AAAACTCTAAACCTACTAAATCCTACACAT	chr2:58468474 – 58468854	381	-

FANCM	NM_020937	50	F: GAAAAAGAGATTTGGTATTTTG<br>R: TAAAATAACCACCAATTTAAAAACTTAATA	chr14:45604667 – 45604833	167	+
BRCA1	NM_007294	55.3	F: ATAGGGGGTTTAAGTGATGTT<br>R: TCAATACCCCCTTCCTAATCC	chr17:41277080 – 41277265	186	-
BRCA2	NM_000059	47.5	F: ATTTAAATATGAGTTGGAGTAAAAAGAAAG<br>R: AAAATTCCTTATCAAACATCCCAA	chr13:32889334 – 32889510	177	+
MLH1	NM_001167619	52.3	F: GGTGATTGGTTGAAGGTATT<br>R: CCAATTCTCAATCATCTCTT	chr3:37034975 – 37035155	181	+
CHEK2	NM_007194	54.6	F: GTGGGTAGAAGAATTTGTTT<br>R: CCTCAATACCTCCTAAAAATTA	chr22:29137910 – 29138261	352	-
MHS2	NM_000251	52.3	F: AGTTTTGGAAGTTGATTGGG<br>R: TCCTCACCTCCTAATTAAAAA	chr2:47630198 – 47630321	124	+
PALB2	NM_024675	55.3	F: AAGAGGAGGATATATATTTGGATT<br>R: ATCCCAATAAACCACCTAAAA	chr16:23652747 – 23652942	196	-
PMS2	NM_001322007	52.3	F: GTATGGTAGAATTAAAGTAAAAGGGGG<br>R: ACCTAAACCAATCAAAACACAC	chr7:6048846 – 6048998	153	-
POLH	NM_001291970	59.6	F: GGAGTTGGGGTTTATTATGA<br>R: CACCTAAACTCAACCTACACA	chr6:43543527 – 43543844	318	+
FANCI	NM_001113378	59.6	F: TAGGATAGGAGGGAAGTTGAAT<br>R: AACAAAAACCTCCTTTCCCC	chr15:89786973 – 89787364	392	+
*KLLN***	NM_001126049	47.5	F: TTAGATAGGTAAAGAATTAAGTATGG<br>R: AAAATTTCACCATATTAATCAAAATAA	chr10:89619479 – 89619611	133	+
*MLH1****	NM_001167619	55.3	F: GGAGTGAAGGAGGTTACGGGTAAGT<br>R: AAAAACGATAAAACCCTATACCTAATCTATC	chr3:37034291 – 37034472	182	+

***All genome coordinates are based on the human genome GRCh37/hg19.**

**** Internal positive control.**

*****Conversion control according to Zymo Research.**

[a]Experimental annealing temperature of primers.

Table S2. Target Sites CpG mapping of each gene in the study.

Gene	Nº CpG sites	CpG sites*
EPCAM	17	cg47596828, cg47596830, cg47596840, cg47596849, cg47596853, cg47596858, cg47596862, cg47596869, cg47596883, cg47596885, cg47596887, cg47596897, cg47596900, cg47596907, cg47596912, cg47596930, cg47596942
BRIP1	16	cg59940970, cg59940975, cg59940988, cg59940997, cg59941017, cg59941051, cg59941053, cg59941056, cg59941067, cg59941110, cg59941133, cg59941136, cg59941187, cg59941191, cg59941201, cg59941202
ATR	4	cg142297977, cg142298019, cg142298045, cg142298054
ATM	31	cg108093323, cg108093335, cg108093338, cg108093359, cg108093371, cg108093373, cg108093386, cg108093395, cg108093402, cg108093413, cg108093419, cg108093436, cg108093439, cg108093451, cg108093457, cg108093461, cg108093467, cg108093476, cg108093485, cg108093505, cg108093520, cg108093529, cg108093532, cg108093538, cg108093560, cg108093567, cg108093572, cg108093579, cg108093584, cg108093590, cg108093598
ERCC3	8	cg128051674, cg128051690, cg128051704, cg128051707, cg128051709, cg128051716, cg128051733, cg128051743
RAD51C	27	cg56769830, cg56769866, cg56769883, cg56769891, cg56769895, cg56769904, cg56769912, cg56769922, cg56769929, cg56769936, cg56769941, cg56769944, cg56769948, cg56769956, cg56769962, cg56769966, cg56769982, cg56770003, cg56770008, cg56770010, cg56770018, cg56770023, cg56770038, cg56770057, cg56770069, cg56770074, cg56770093
FANCF	12	cg22647545, cg22647600, cg22647610, cg22647618, cg22647622, cg22647643, cg22647649, cg22647651, cg22647661, cg22647745, cg22647764, cg22647774
FANCL	19	cg58468506, cg58468513, cg58468519, cg58468523, cg58468527, cg58468530, cg58468532, cg58468537, cg58468555, cg58468576, cg58468578, cg58468582, cg58468599, cg58468618, cg58468625, cg58468632, cg58468652, cg58468737, cg58468823
FANCM	2	cg45604787, cg45604794
BRCA1	9	cg41277106, cg 41277115, cg41277117, cg41277153, cg41277160, cg41277174, cg41277187, cg41277214, cg41277232
BRCA2	4	cg32889434, cg32889461, cg32889470, cg32889484

MLH1	13	cg37034997, cg37035012, cg37035032, cg37035043, cg37035047, cg37035063, cg37035066, cg37035074, cg37035090, cg37035095, cg37035097, cg37035100, cg37035117
CHEK2	24	cg29137950, cg29137964, cg29137975, cg29137985, cg29137993, cg29138031, cg29138052, cg29138062, cg29138075, cg29138078, cg29138091, cg29138102, cg29138115, cg29138134, cg29138158, cg29138168, cg29138176, cg29138179, cg29138185, cg29138202, cg29138216, cg29138219, cg29138234, cg29138238
MHS2	10	cg47630224, cg47630227, cg47630233, cg47630237, cg47630240, cg47630244, cg47630251, cg47630265, cg47630292, cg47630294
PALB2	13	cg23652769, cg23652783, cg23652787, cg23652797, cg23652818, cg23652838, cg23652857, cg23652860, cg23652876, cg23652906, cg23652911, cg23652916, cg23652918
PMS2	13	cg6048869, cg6048876, cg6048884, cg6048888, cg6048892, cg6048904, cg6048918, cg6048925, cg6048928, cg6048940, cg6048951, cg6048966, cg6048968
POLH	40	cg43543548, cg43543554, cg43543571, cg43543578, cg43543580, cg43543596, cg43543608, cg43543613, cg43543615, cg43543618, cg43543624, cg43543642, cg43543650, cg43543657, cg43543669, cg43543672, cg43543682, cg43543691, cg43543696, cg43543698, cg43543701, cg43543705, cg43543708, cg43543733, cg43543747, cg43543749, cg43543763, cg43543766, cg43543771, cg43543775, cg43543777, cg43543781, cg43543786, cg43543794, cg43543798, cg43543800, cg43543804, cg43543806, cg43543815, cg43543822
FANCI	38	cg89786999, cg89787007, cg89787009, cg89787018, cg89787032, cg89787034, cg89787036, cg89787045, cg89787048, cg89787058, cg89787060, cg89787066, cg89787077, cg89787087, cg89787103, cg89787109, cg89787113, cg89787120, cg89787150, cg89787154, cg89787164, cg89787168, cg89787171, cg89787182, cg89787184, cg89787190, cg89787205, cg89787212, cg89787223, cg89787226, cg89787238, cg89787260, cg89787272, cg89787288, cg89787293, cg89787295, cg89787304, cg89787335
*KLLN***	8	cg89619506, cg89619510, cg89619512, cg89619522, cg89619550, cg89619555, cg89619566, cg89619580
*MLH1****	22	cg37034316, cg37034324, cg37034330, cg37034344, cg37034346, cg37034348, cg37034352, cg37034355, cg37034359, cg37034369, cg37034373, cg37034382, cg37034394, cg37034402, cg37034409, cg37034412, cg37034414, cg37034418, cg37034420, cg37034430, cg37034438, cg37034441

***All genome sites CpG are based on the human genome GRCh37/hg19.**

Table S3. Z-score values (Methylation index) in the promoter regions of the studied genes.

Gene	CGI	Chromosome	Genomic coordinate	Mean Z-score		p*
				Controls (N=156)	Patients (N=231)	
KLLN_Cont	Shelf	10	89619479 – 89619611	6.06E-15	-0.331	0.001
FANCF	Shore	11	22647502 – 22647882	5.65E-16	0.223	0.641
ATM	Island	11	108093274 – 108093619	8.18E-17	-0.145	0.104
BRCA2	Island	13	32889334 – 32889510	-3.67E-16	-0.221	0.006
FANCM	Shore	14	45604667 – 45604833	2.07E-16	-0.329	0.005
FANCI	Island	15	89786973 – 89787364	-2.22E-16	-0.122	0.114
PALB2	Island	16	23652747 – 23652942	-3.03E-16	0.047	0.889
BRCA1	Shore	17	41277080 – 41277265	1.30E-16	-0.035	0.247
RAD51C	Island	17	56769721 – 56770125	-1.17E-16	0.132	0.048
BRIP1	Island	17	59940949 – 59941237	-4.74E-16	-0.091	0.176
EPCAM	Island	2	47596802 – 47596963	-3.74E-16	0.326	0.017
MSH2	Island	2	47630198 – 47630321	-2.47E-16	-0.045	0.575
FANCL	Island	2	58468474 – 58468854	3.98E-17	-0.391	0.007
ERCC3	Island	2	128051655 – 128051766	-3.28E-16	-0.282	<0.001
CHEK2	Island	22	29137910 – 29138261	-4.48E-16	-0.412	<0.001
MLH1_Cont	Shore	3	37034291 – 37034472	9.86E-17	-0.613	<0.001
MLH1	Island	3	37034975 – 37035155	-3.45E-16	-0.468	0.001
ATR	Shore	3	142297947 –142298077	1.07E-16	-0.172	0.077
POLH	Island	6	43543527 – 43543844	8.11E-17	0.131	0.217
PMS2	Island	7	6048846 – 6048998	-1.01E-15	-0.434	<0.001

***The value of *p*<0.05 is considered significant. Wilcoxon-Mann-Whitney statistical test.**

Table S4. Z-score value (Methylation index) of sites 36 CpG hypermethylated in study

Gene	Chromosome	CpG site	Mean Z-score: Controls (N=156)	Mean Z-score: Patients (N=231)	*P**	FDR q-value**
ATM	11	cg108093323	1.54E-10	0.095	0.005	0.001
ATM	11	cg108093386	-3.21E-11	0.176	0.023	7.49E-05
ATM	11	cg108093485	-1.41E-10	0.007	0.036	0.039
ATM	11	cg108093590	-1.92E-10	0.069	0.002	7.49E-04
FANCI	**15**	**cg89786999**	**7.69E-11**	**1.260**	**0.001**	**1.10E-05**
PALB2	**16**	**cg23652916**	**1.92E-11**	**2.265**	**1.19E-07**	**3.34E-09**
RAD51C	17	cg56769883	1.03E-10	0.003	0.015	0.003
RAD51C	17	cg56769891	1.67E-10	0.063	0.010	0.002
RAD51C	17	cg56769895	7.69E-11	0.119	0.034	0.006
RAD51C	17	cg56770018	7.69E-11	0.052	0.008	0.001
BRIP1	17	cg59940988	1.03E-10	0.264	0.022	0.004
CHEK2	22	cg29138238	6.41E-11	0.253	0.012	0.002
EPCAM	**2**	**cg47596828**	**-2.56E-11**	**1.073**	**7.74E-09**	**4.33E-10**
EPCAM	2	cg47596830	-1.92E-11	0.402	0.011	0.002
EPCAM	2	cg47596849	-4.49E-11	0.261	0.041	0.007
EPCAM	2	cg47596858	1.92E-11	0.363	0.007	0.001
EPCAM	2	cg47596862	1.92E-11	0.346	0.014	0.002
EPCAM	2	cg47596869	-1.92E-11	0.216	0.040	0.009
EPCAM	2	cg47596887	3.85E-11	0.305	0.028	0.005
EPCAM	2	cg47596897	1.92E-11	0.326	0.018	0.003
EPCAM	2	cg47596912	1.92E-11	0.332	0.029	0.004
MSH2	**2**	**cg47630224**	**-6.41E-12**	**2.952**	**1.99E-10**	**2.23E-11**
POLH	6	cg43543596	1.47E-10	0.210	0.025	0.005
POLH	6	cg43543608	-6.41E-11	0.024	0.001	4.53E-04
POLH	6	cg43543613	-2.56E-11	0.125	0.040	0.008

						0.002
POLH	6	cg43543615	-1.73E-10	0.541	0.014	
POLH	6	cg43543624	-2.12E-10	0.246	0.041	0.020
POLH	6	cg43543642	3.21E-11	0.085	0.012	0.002
POLH	6	cg43543657	-1.28E-11	0.095	0.046	0.007
POLH	6	cg43543669	2.63E-10	0.077	0.008	0.001
POLH	6	cg43543705	-1.92E-11	0.055	0.022	7.49E-05
POLH	6	cg43543747	-1.67E-10	0.027	0.011	0.002
POLH	6	cg43543749	-8.33E-11	0.048	0.005	0.001
POLH	6	cg43543763	1.09E-10	0.086	0.012	0.002
POLH	6	cg43543766	-1.54E-10	0.114	0.038	0.006
POLH	6	cg43543794	-1.09E-10	0.204	0.019	0.003

***The value of *p*<0.05 is considered significant. Wilcoxon-Mann-Whitney statistical test with Bonferroni correction.**

**** Methylation of CpG sites meeting the false discovery rate (FDR) criteria (q <0.05).**

Table S5. Z-score value (Methylation index) of sites CpG across the promoter region of 4 genes suppress tumor

Gene Promoter	Strand	CGI	CpG site	Mean Z-score Controls (N=156)	Mean Z-score Patients (N=231)	*p**
***PALB2* Promoter Region**	-	Island	cg23652769	-1.09E-10	-0.047	0.401
	-	Island	cg23652783	4.49E-11	-0.180	0.014
	-	Island	cg23652787	-1.92E-11	-0.146	0.096
	-	Island	cg23652797	-6.41E-12	-0.312	0.471
	-	Island	cg23652818	-4.49E-11	-0.034	0.760
	-	Island	cg23652838	-3.85E-11	-0.020	0.426
	-	Island	cg23652857	-1.28E-11	-0.175	0.029
	-	Island	cg23652860	-6.41E-12	-0.331	0.068
	-	Island	cg23652876	-2.05E-10	-0.191	0.014
	-	Island	cg23652906	7.05E-11	-0.335	0.216
	-	Island	cg23652911	1.28E-11	-0.143	0.111
	**-**	**Island**	**cg23652916**	**1.92E-11**	**2.265**	**1.19E-07**
	-	Island	cg23652918	-6.41E-12	-0.067	0.614
***EPCAM* Promoter Region**	**+**	**Island**	**cg47596828**	**-2.56E-11**	**1.073**	**7.74E-09**
	+	Island	cg47596830	-1.92E-11	0.402	0.011
	+	Island	cg47596840	1.28E-11	0.263	0.298
	+	Island	cg47596849	-4.49E-11	0.261	0.041
	+	Island	cg47596853	5.77E-11	0.215	0.344
	+	Island	cg47596858	1.92E-11	0.363	0.007
	+	Island	cg47596862	1.92E-11	0.346	0.014
	+	Island	cg47596869	-1.92E-11	0.216	0.040
	+	Island	cg47596883	1.28E-11	0.206	0.110
	+	Island	cg47596885	-1.92E-11	0.226	0.249

	+	Island	cg47596887	3.85E-11	0.305	0.028
	+	Island	cg47596897	1.92E-11	0.326	0.018
	+	Island	cg47596900	-3.85E-11	0.199	0.116
	+	Island	cg47596907	-1.92E-11	0.191	0.265
	+	Island	cg47596912	1.92E-11	0.332	0.029
	+	Island	cg47596930	3.85E-11	0.185	0.110
	+	Island	cg47596942	1.28E-11	0.179	0.185
***MHS2* Promoter Region**	**+**	**Island**	**cg47630224**	**-6.41E-12**	**2.952**	**1.99E-10**
	+	Island	cg47630227	-6.41E-12	-0.195	0.032
	+	Island	cg47630233	-1.28E-11	-0.503	0.004
	+	Island	cg47630237	6.41E-12	-0.322	0.247
	+	Island	cg47630240	6.41E-12	-0.435	0.006
	+	Island	cg47630244	1.28E-11	-0.194	0.030
	+	Island	cg47630251	-4.49E-11	-0.260	0.009
	+	Island	cg47630265	-4.49E-11	-0.129	0.045
	+	Island	cg47630292	-9.25E-18	-0.305	0.430
	+	Island	cg47630294	6.41E-12	-0.385	0.015
***FANCI* Promoter Region**	**+**	**Island**	**cg89786999**	**7.69E-11**	**1.260**	**0.001**
	+	Island	cg89787007	-1.09E-10	0.011	0.708
	+	Island	cg89787009	2.05E-10	0.007	0.329
	+	Island	cg89787018	-5.77E-11	0.022	0.044
	+	Island	cg89787032	1.35E-10	-0.110	0.017
	+	Island	cg89787034	-8.33E-11	-0.176	0.711
	+	Island	cg89787036	-5.13E-11	0.033	0.205
	+	Island	cg89787045	3.21E-11	-0.047	0.043
	+	Island	cg89787048	-1.92E-11	0.044	0.090
	+	Island	cg89787058	1.28E-11	-0.042	0.094
	+	Island	cg89787060	1.15E-10	-0.097	0.061
	+	Island	cg89787066	2.56E-11	-0.018	0.077

+	Island	cg89787077	3.85E-11	0.102	0.546
+	Island	cg89787087	-6.41E-12	-0.018	0.181
+	Island	cg89787103	-1.15E-10	-0.075	0.022
+	Island	cg89787109	-8.33E-11	-0.234	0.205
+	Island	cg89787113	7.69E-11	-0.343	0.026
+	Island	cg89787120	2.31E-10	-0.217	0.108
+	Island	cg89787150	2.56E-11	-0.092	0.238
+	Island	cg89787154	-2.56E-11	-0.127	0.455
+	Island	cg89787164	-2.56E-11	0.028	0.527
+	Island	cg89787168	1.99E-10	-0.019	0.005
+	Island	cg89787171	-2.31E-10	0.176	0.093
+	Island	cg89787182	5.13E-11	-0.272	0.225
+	Island	cg89787184	-5.77E-11	-0.112	0.015
+	Island	cg89787190	2.05E-10	-0.106	0.991
+	Island	cg89787205	2.12E-10	-0.202	0.167
+	Island	cg89787212	-1.86E-10	-0.163	0.005
+	Island	cg89787223	1.99E-10	-0.091	0.064
+	Island	cg89787226	-1.28E-10	-0.176	0.162
+	Island	cg89787238	-1.86E-10	-0.250	0.001
+	Island	cg89787260	-4.27E-18	-0.225	0.621
+	Island	cg89787272	-2.56E-11	-0.258	0.026
+	Island	cg89787288	-1.09E-10	-0.176	0.017
+	Island	cg89787293	1.09E-10	-0.222	0.194
+	Island	cg89787295	-4.49E-11	-0.219	0.657
+	Island	cg89787304	-1.47E-10	-0.037	0.127
+	Island	cg89787335	-1.28E-11	0.023	0.532

***The value of $p<0.05$ is considered significant. Wilcoxon-Mann-Whitney statistical test with Bonferroni correction.**

Table S6. The association of the methylation percentage per CpG site on *EPCAM, FANCI, MSH2,* and *PALB2* with the risk of breast cancer

CpG Position	Univariate analysis			Multivariate analysis		
	OR	95% CI	*P* value	OR*	95% CI	*P* value
cg47596828_*EPCAM*	1.66	1.42 - 1.93	<0.001	1.84	1.46 - 2.32	<0.001
cg89786999_*FANCI*	1.70	1.40 - 2.08	<0.001	1.65	1.24 - 2.20	0.001
cg47630224_*MSH2*	4.57	2.66 - 7.86	<0.001	4.17	2.05 - 8.48	<0.001
cg23652916_*PALB2*	2.88	2.01 - 4.13	<0.001	2.83	1.71 - 4.66	<0.001

Odds ratios and 95% confidence intervals per methylation percent increment.
***ORs adjusted for age, age at menarche, BMI, family history of cancer, and depth of sequencing.**

Table S7. The association of the methylation Z-Score per CpG site on *EPCAM, FANCI, MSH2*, and *PALB2* with the risk of breast cancer

CpG Position	Univariate analysis			Multivariate analysis		
	OR	95% CI	*P* value	OR*	95% CI	*P* value
cg47596828_*EPCAM*	1.86	1.54 - 2.24	<0.001	2.11	1.59 - 2.81	<0.001
cg89786999_*FANCI*	1.52	1.30 - 1.77	<0.001	1.48	1.18 - 1.85	0.001
cg47630224_*MSH2*	1.58	1.34 - 1.85	<0.001	1.53	1.24 - 1.90	<0.001
cg23652916_*PALB2*	1.56	1.34 - 1.82	<0.001	1.55	1.25 - 1.91	<0.001

Odds ratios and 95% confidence intervals per methylation percent increment.
***ORs adjusted for age, age at menarche, BMI, family history of cancer, and depth of sequencing.**

Table S8. The association of the methylation percentage quartiles per CpG site on *EPCAM* with the risk of breast cancer

CpG Position	Univariate analysis			Multivariate analysis		
	OR	95% CI	*P* value	OR*	95% CI	*P* value
cg47596828_*EPCAM*_Q1 n=62 (0.9966777 - 2.655827 %)	1.00			1.00		
cg47596828_*EPCAM*_Q2 n=82 (2.660017 - 3.488372 %)	1.87	0.95 - 3.67	0.069	1.32	0.51 - 3.40	0.571
cg47596828_*EPCAM*_Q3 n=72 (3.489362 - 4.335766 %)	1.43	0.72 - 2.87	0.307	2.02	0.72 - 5.69	0.185
cg47596828_*EPCAM*_Q4 n=171 (4.354469 - 15.8288 %)	5.74	3.07 - 10.74	<0.001	6.47	2.51 - 16.71	<0.001

Odds ratios and 95% confidence intervals per methylation percent increment.
***ORs adjusted for age, age at menarche, BMI, family history of cancer, and depth of sequencing.**

Table S9. The association of the methylation percentage quartiles per CpG site on *FANCI* with the risk of breast cancer

CpG Position	Univariate analysis			Multivariate analysis		
	OR	95% CI	*P* value	OR*	95% CI	*P* value
cg89786999_*FANCI*_Q1 n=143 (0 %)	1.00			1.00		
cg89786999_*FANCI*_Q2 n=18 (0.1193317 - 0.3205128 %)	0.56	0.21 - 1.53	0.259	3.53	0.67 - 18.52	0.136
cg89786999_*FANCI*_Q3 n=75 (0.3246753 - .877193 %)	0.81	0.47 - 1.42	0.470	2.14	0.89 - 5.15	0.090
cg89786999_*FANCI*_Q4 n=151 (0.8791209 - 10.66667 %)	2.53	1.55 - 4.13	<0.001	2.69	1.31 - 5.52	0.007

Odds ratios and 95% confidence intervals per methylation percent increment.

***ORs adjusted for age, age at menarche, BMI, family history of cancer, and depth of sequencing.**

Table S10. The association of the methylation percentage quartiles per CpG site on *MSH2* with the risk of breast cancer

CpG Position	Univariate analysis			Multivariate analysis		
	OR	95% CI	*P* value	OR*	95% CI	*P* value
cg47630224_*MSH2*_Q1 n=74 (0 - 0.3755869 %)	1.00			1.00		
cg47630224_*MSH2*_Q2 n=60 (0.378519 - 0.5548595 %)	0.60	0.30 - 1.21	0.153	0.96	0.35 - 2.61	0.932
cg47630224_*MSH2*_Q3 n=77 (0.5566379 - 0.7697242 %)	1.09	0.57 - 2.06	0.801	1.72	0.66 - 4.50	0.268
cg47630224_*MSH2*_Q4 n=176 (0.7710558 - 5.922551 %)	3.91	2.19 - 6.98	<0.001	4.74	2.09 - 10.74	<0.001

Odds ratios and 95% confidence intervals per methylation percent increment.
***ORs adjusted for age, age at menarche, BMI, family history of cancer, and depth of sequencing.**

Table S11. The association of the methylation percentage quartiles per CpG site on *PALB2* with the risk of breast cancer

CpG Position	Univariate analysis			Multivariate analysis		
	OR	95% CI	*P* value	OR*	95% CI	*P* value
cg23652916_*PALB2*_Q1 n=72 (0 - 0.3889159 %)	1.00			1.00		
cg23652916_*PALB2*_Q2 n=65 (.03919861 - 0.6045519 %)	0.79	0.40 - 1.55	0.491	0.85	0.31 - 2.31	0.745
cg23652916_*PALB2*_Q3 n=89 (0.6060606 - 0.9892473 %)	1.52	0.81 - 2.83	0.192	1.98	0.79 - 4.93	0.143
cg23652916_*PALB2*_Q4 n=161 (0.990099 - 7.207207 %)	3.70	2.05 - 6.65	<0.001	2.85	1.19 - 6.80	0.018

Odds ratios and 95% confidence intervals per methylation percent increment.

***ORs adjusted for age, age at menarche, BMI, family history of cancer, and depth of sequencing.**

***Quartiles based on control´s distribution.**

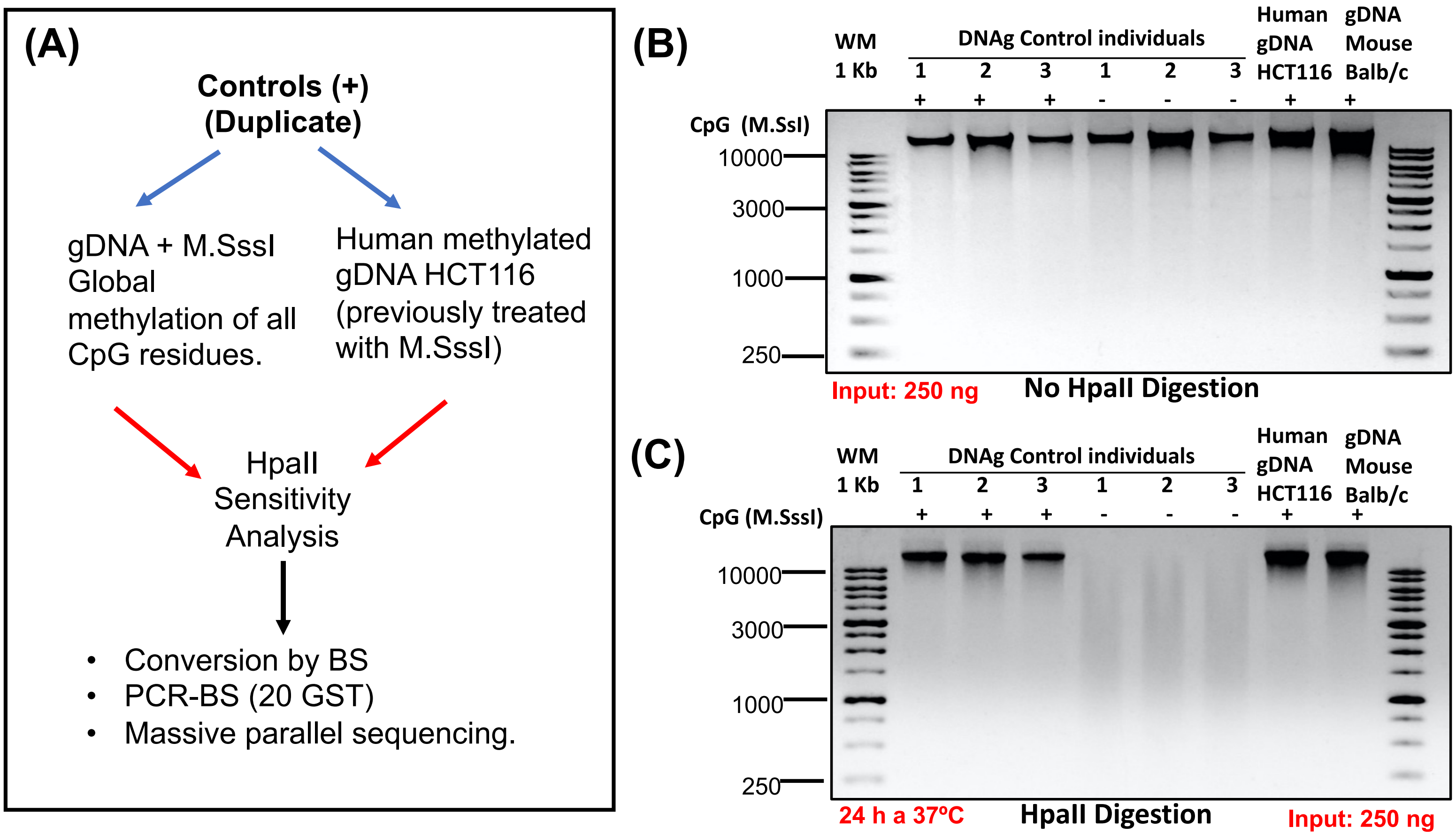

Figure S1. HpaII sensitivity assay to evaluate M.SssI (DNA methyltransferase) activity. A) Validated positive controls used in this study. Experimental strategy. B) gDNA of control individuals with (+) or without (-) M.SssI treatment. HCT116 and Mouse Balb/c are commercial methylated controls. C) HpaII digestion. The M.SssI methylated gDNA controls (+) and commercial methylated controls resist the enzymatic activity of the HpaII. gDNA controls without M. SssI treatment showed fully enzymatic digestion.

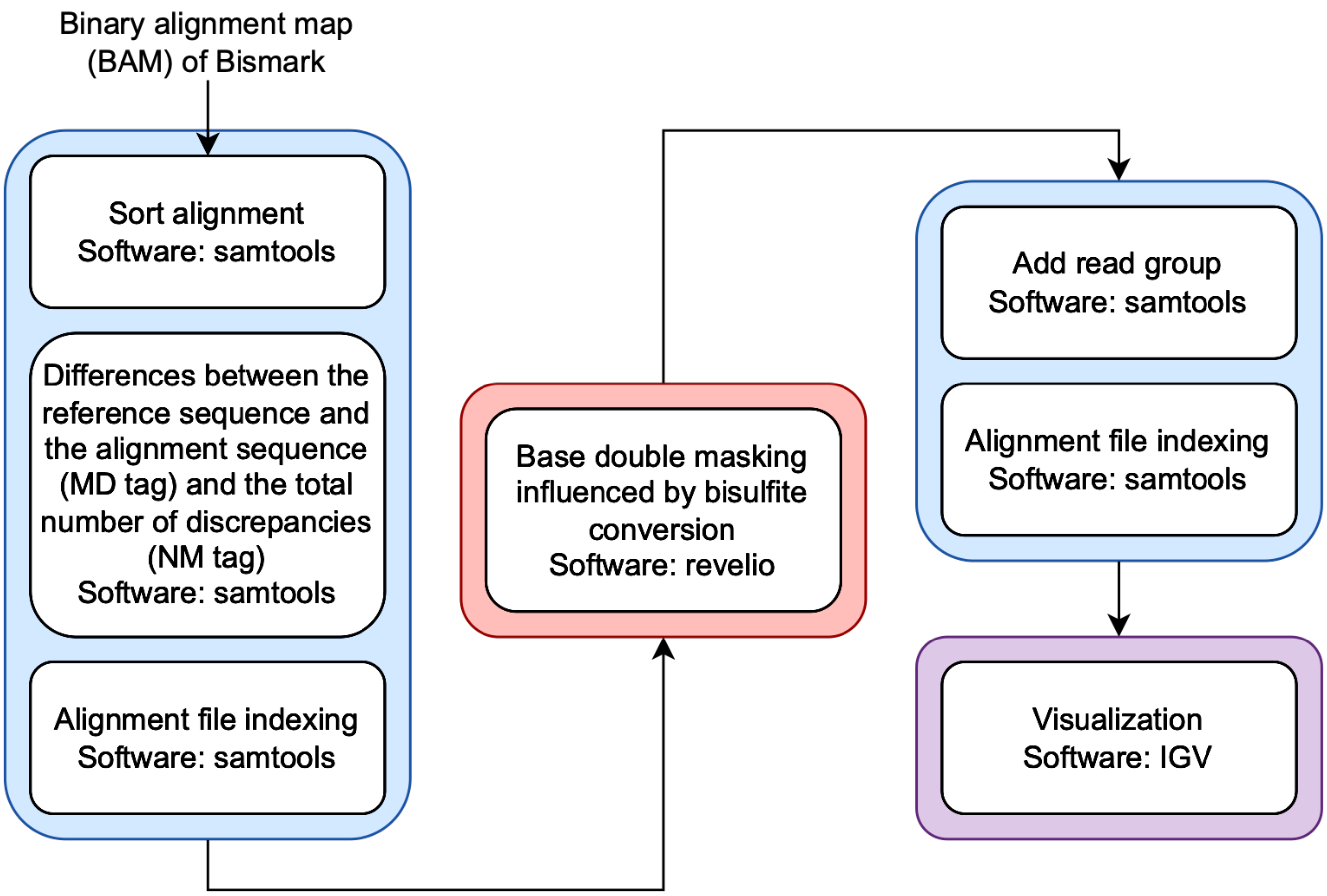

Figure S2. Algorithm implemented for the detection of Single Nucleotide Polymorphisms: Revelio software.

In the following figures (S3-S7), representative Integrative Genomics Viewer (IGV) images are provided for patients with hypermethylated sites at the gene positions: cg23652916-*PALB2*, cg89786999-*FANCI*, cg47630224-*MSH2*, and cg47596828-*EPCAM*, in comparison with controls without hypermethylation at the respective sites. For both groups, a comparison was performed between raw methylation data and the Revelio software. Revelio utilizes a double-masking approach with a Bayesian method for sodium bisulfite-converted sequences, providing clean conversion reads and determining the presence of single nucleotide polymorphisms (SNPs) in the analysis regions.

(A) Patients with hypermethylated cg47596828 site in *EPCAM*

Raw methylation data | **Revelio software**

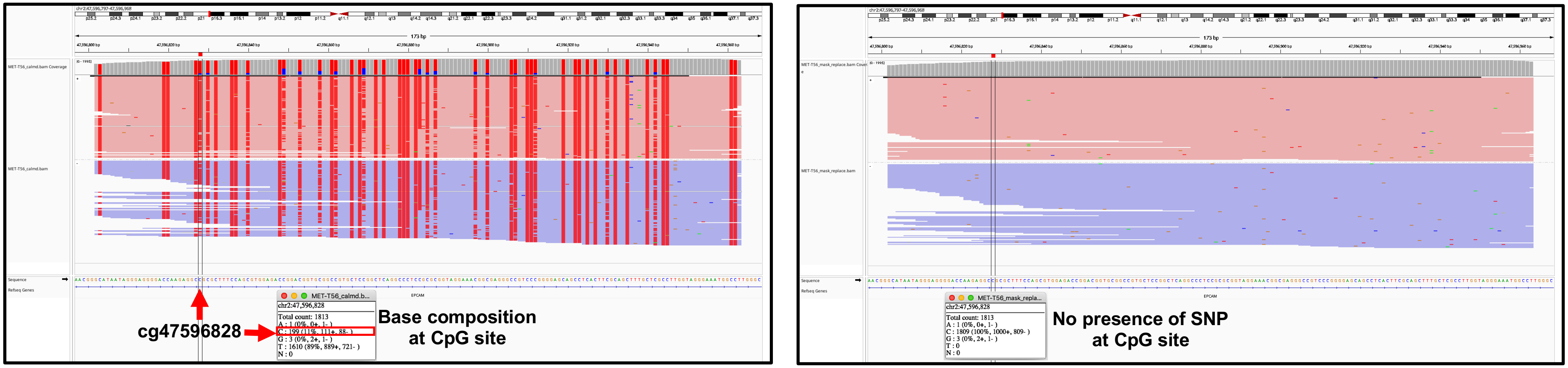

Patient MET-T56. Depth site: 1813X. % Meth: **11.1%**

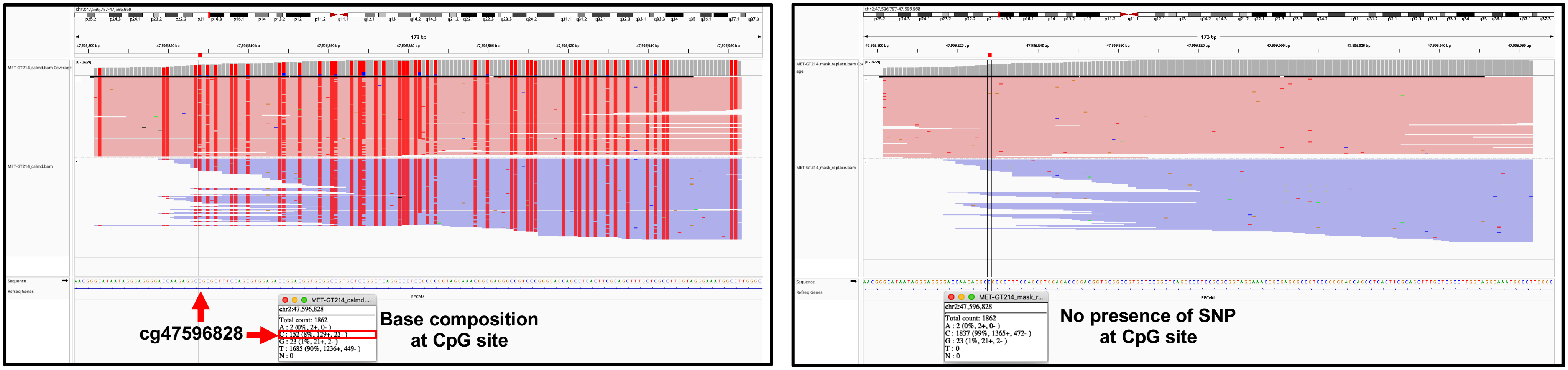

Patient MET-GT214. Depth site: 1862X. % Meth: **8.5%**

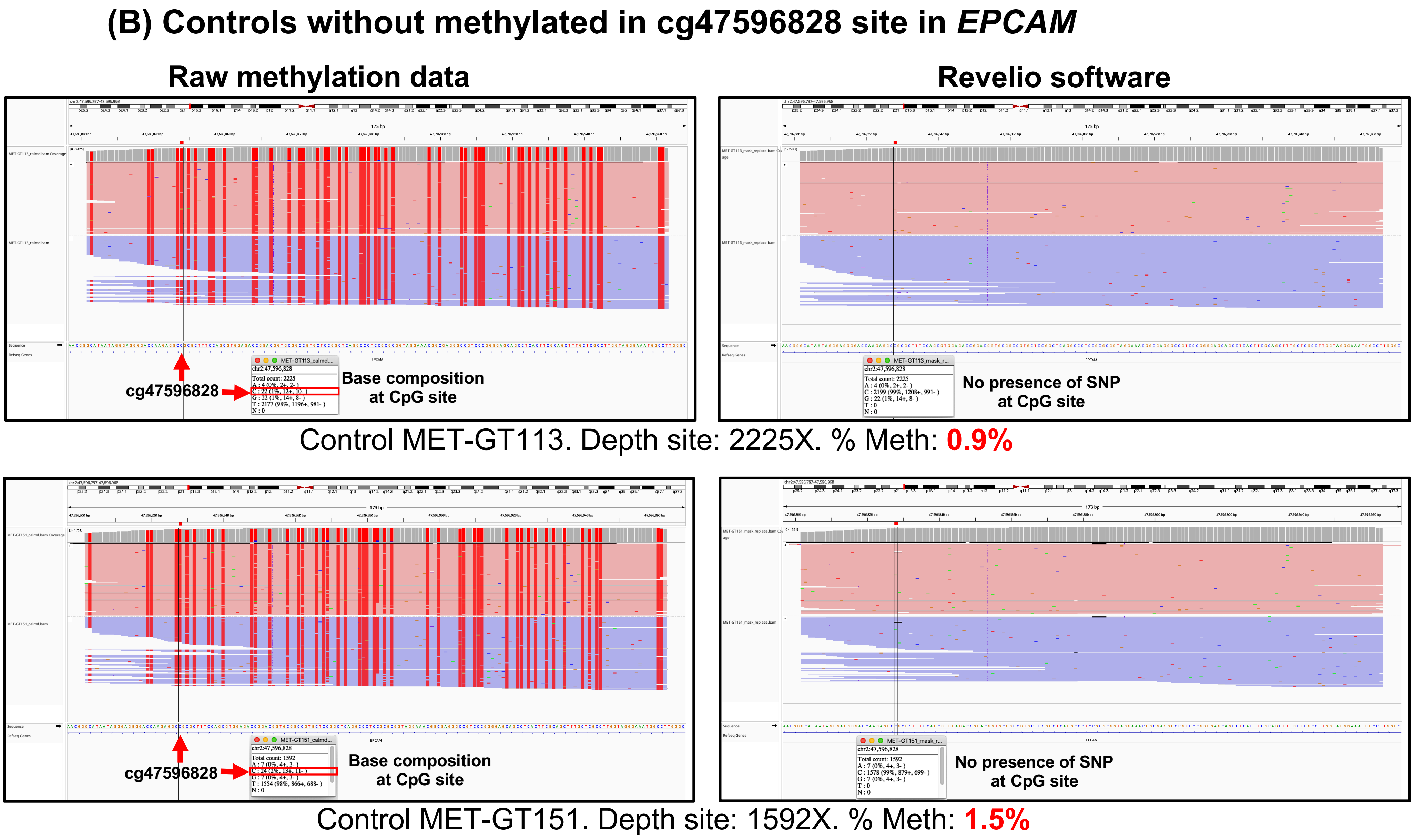

Figure S3. *EPCAM* Promoter Region. **(A)** Two representative IGV images are presented for patients with hypermethylation at the cg47596828 site of *EPCAM*. **(B)** Two representative IGV images are illustrated for controls without hypermethylation at the site. In the left panel, sequences with raw methylation data (treated with sodium bisulfite) are visualized. The C>T change in the sense strand (*EPCAM* gene location with positive polarity) is observed in red due to bisulfite conversion at unmethylated sites. In this case, cytosines resistant to treatment are considered methylated (blue bars). In the right panel, sequences were processed with the Revelio software, enabling the exclusion of SNP presence at the studied methylation site.

(A) Patients with hypermethylated cg47630224 site in *MSH2*

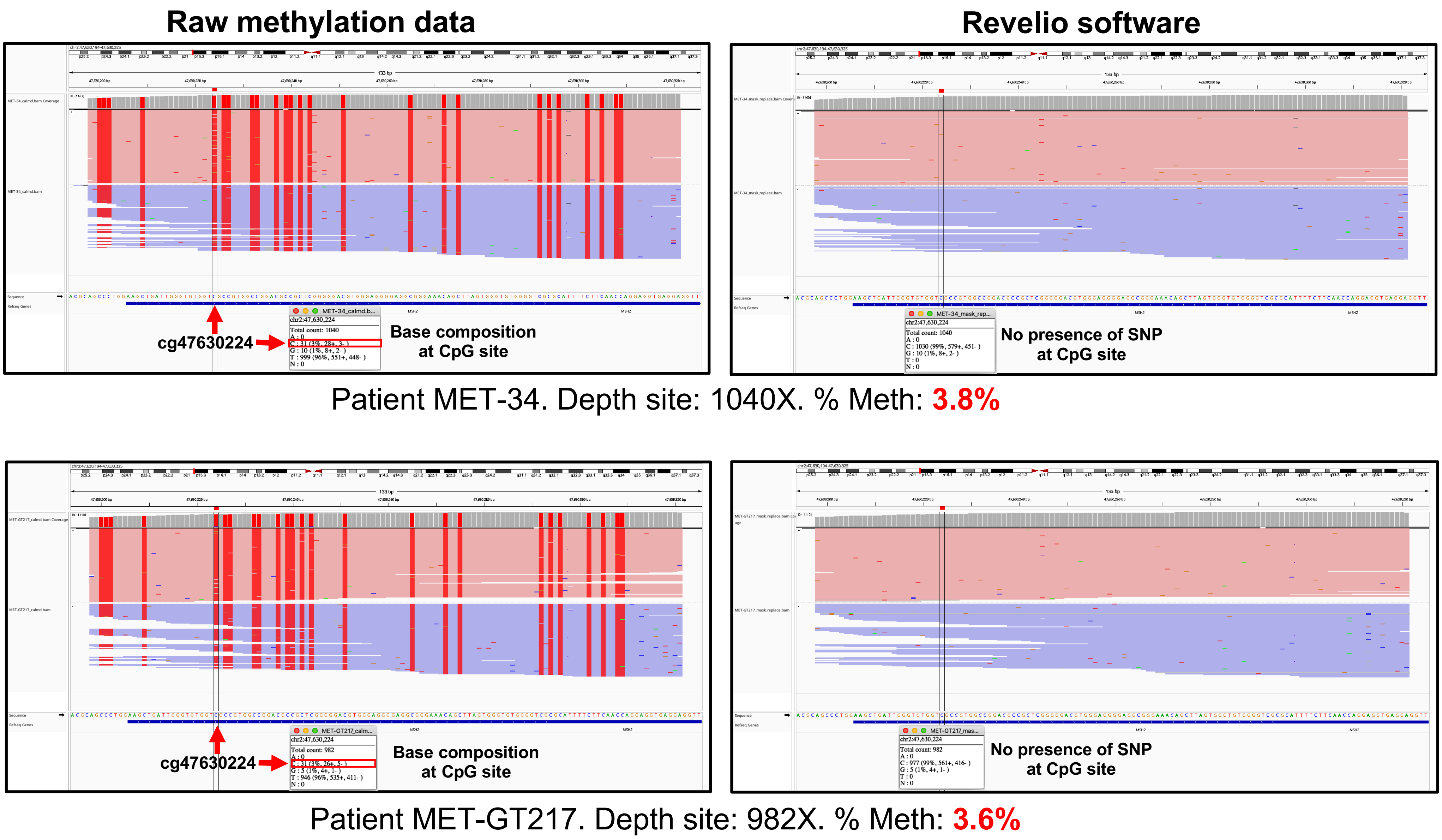

(B) Controls without methylated in cg47630224 site in *MSH2*

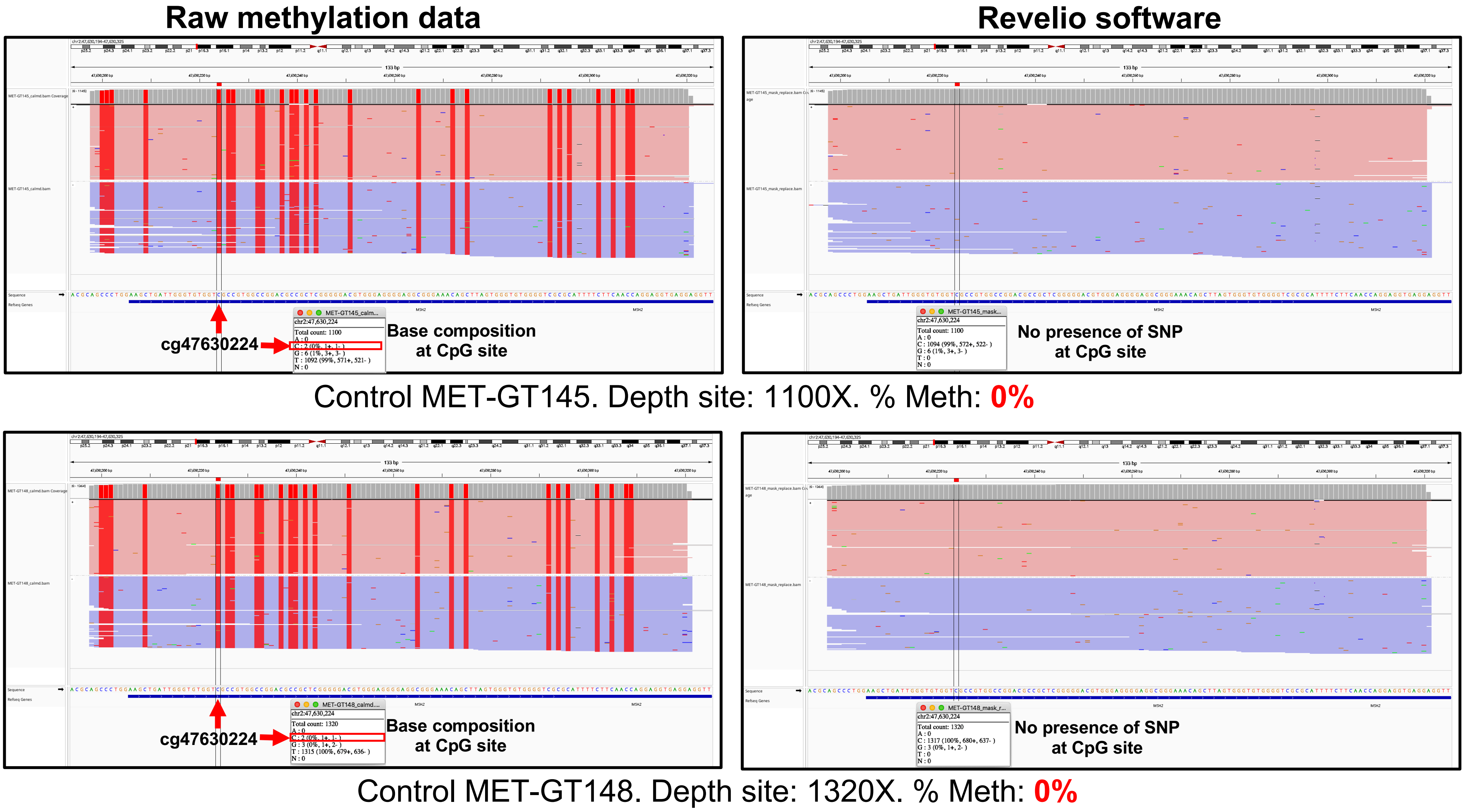

Figure S4. *MSH2* Promoter Region Sequencing. (A) Two representative IGV images are displayed for patients with hypermethylation at the cg47630224 site of *MSH2*. **(B)** Two representative IGV images are illustrated for controls without hypermethylation at the site. In the left panel, sequences with raw methylation data (treated with sodium bisulfite) are visualized. The C>T change in the sense strand (*MSH2* gene location with positive polarity) is observed in red due to bisulfite conversion at unmethylated sites. In this case, cytosines resistant to treatment are considered methylated (blue bars). In the right panel, sequences were processed with the Revelio software, enabling the exclusion of SNP presence at the studied methylation site.

(A) Patients with hypermethylated cg23652916 site in *PALB2*

Raw methylation data | **Revelio software**

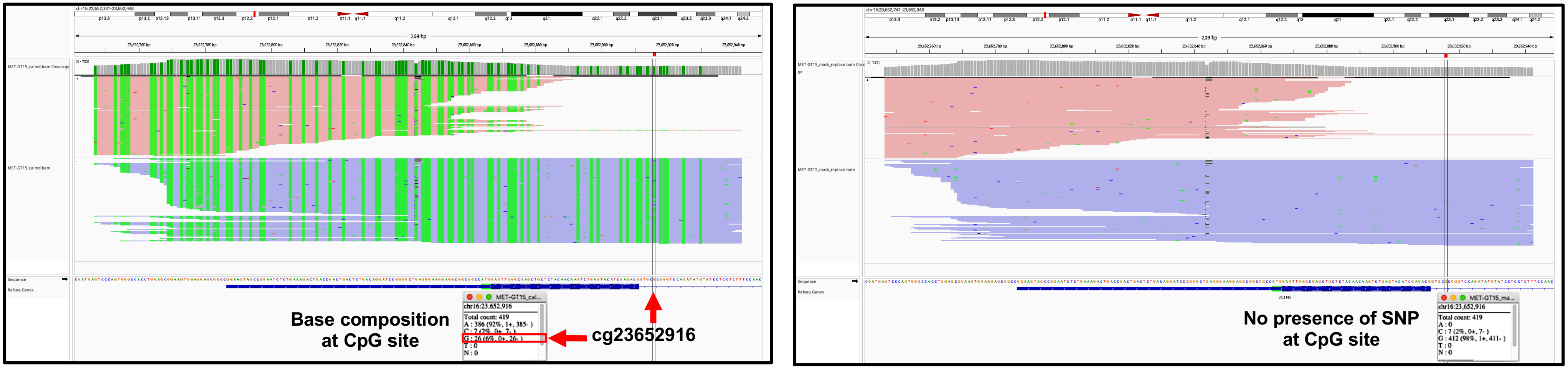

Patient MET-GT15. Depth site: 419X. % Meth: **6.3%**

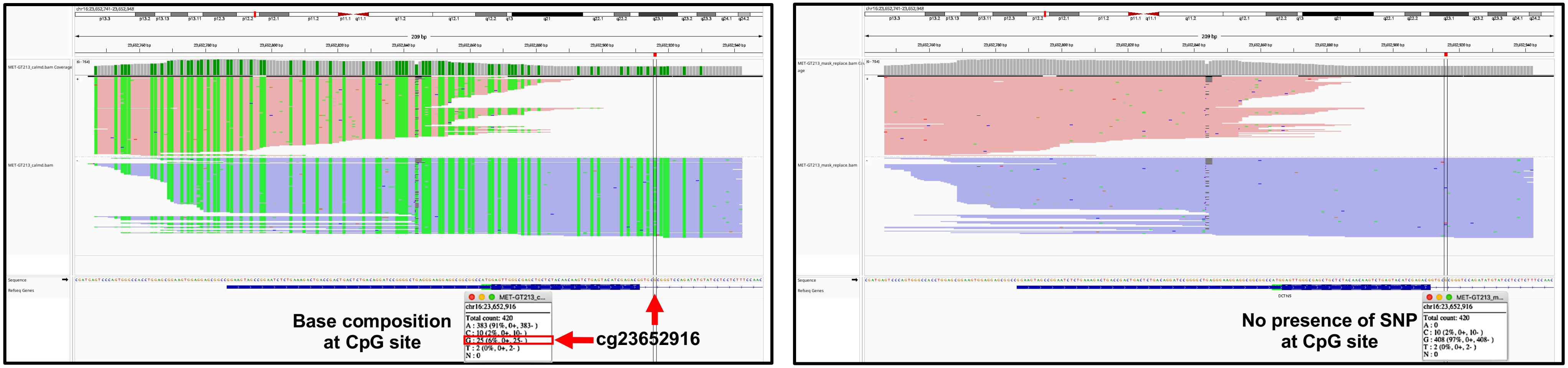

Patient MET-GT213. Depth site: 420X. % Meth: **6%**

(B) Controls without methylated in cg23652916 site of *PALB2*

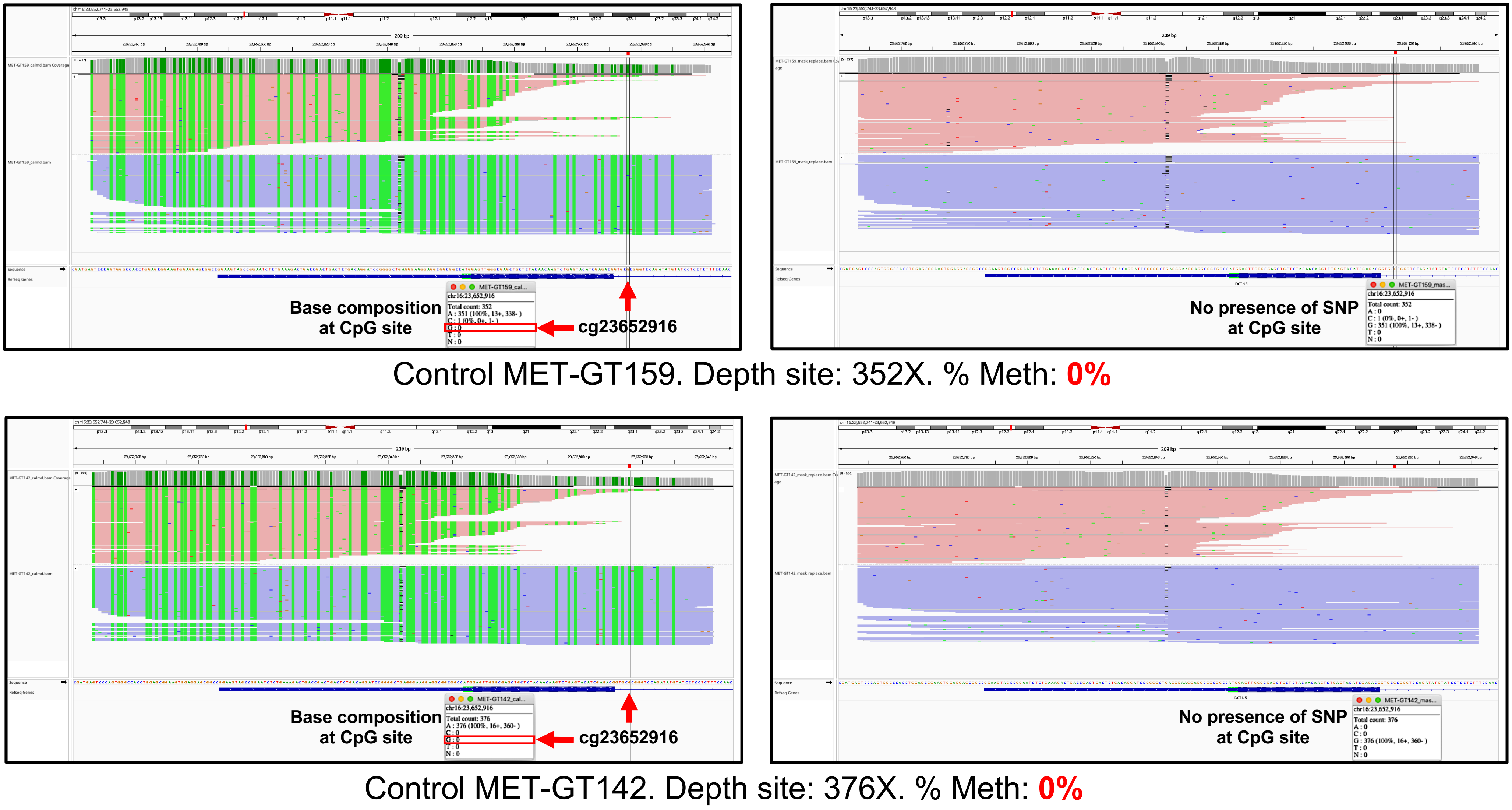

Figure S5. *PALB2* promoter region sequencing. (A) Two representative IGV images are displayed for patients with hypermethylation at the cg23652916 site of *PALB2*. **(B)** Two representative IGV images are illustrated for controls without hypermethylation at the site. In the left panel, sequences with raw methylation data (treated with sodium bisulfite) are visualized. The G>A change in the antisense strand (*PALB2* gene location with negative polarity) is observed in green due to bisulfite conversion at unmethylated sites. In this case, guanines resistant to treatment are considered methylated, as they correspond to cytosines in the complementary strand. In the right panel, sequences were processed with the Revelio software, which allowed for the exclusion of SNP presence at the methylation site under study.

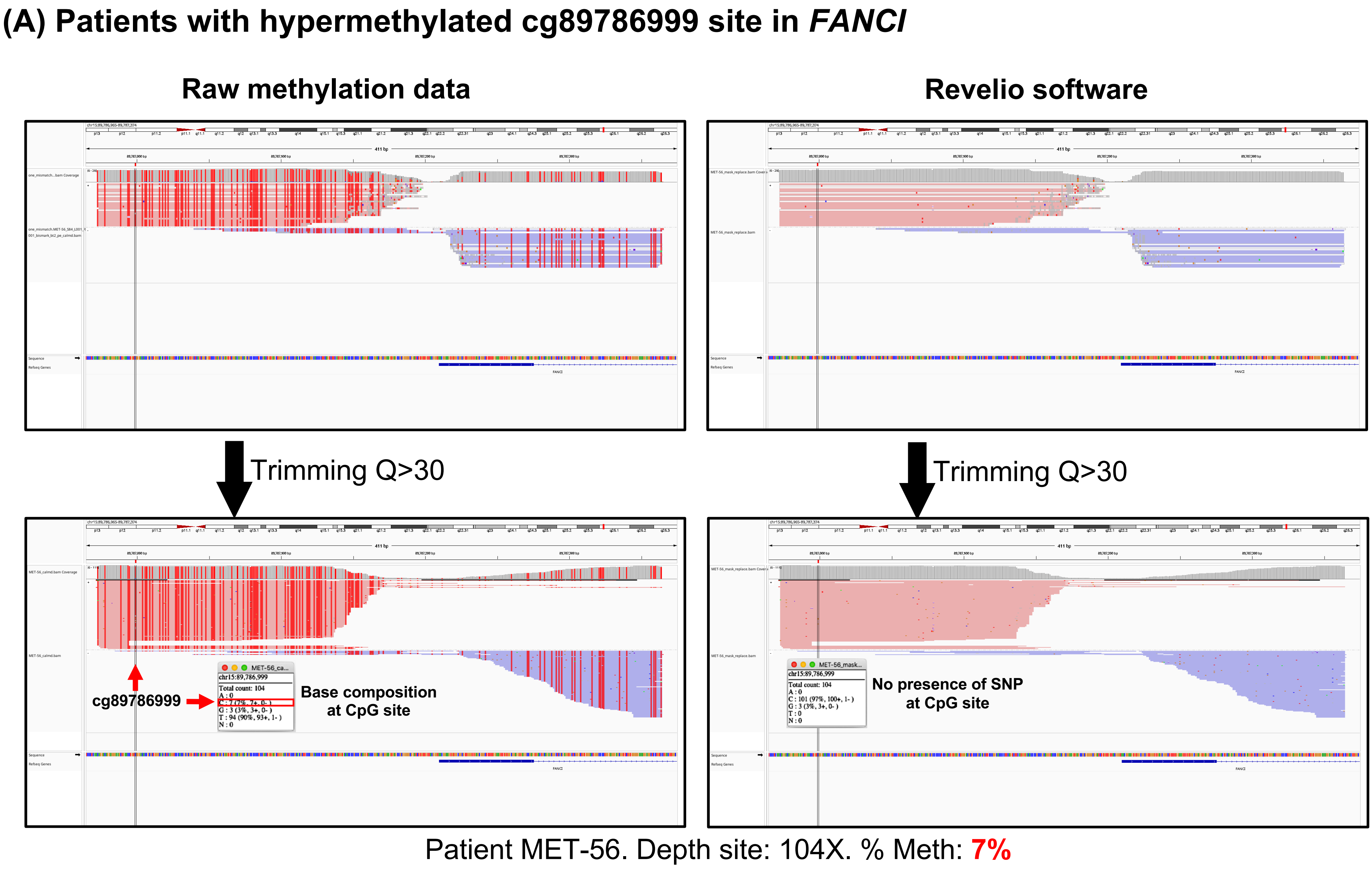
(A) Patients with hypermethylated cg89786999 site in FANCI
Raw methylation data
Revelio software
Trimming Q>30
Trimming Q>30
cg89786999
chr15:89,786,999
Total count: 104
A : 0
C : 7 (7%, 7+, 0-)
G : 3 (3%, 3+, 0-)
T : 94 (90%, 93+, 1-)
N : 0
Base composition at CpG site
chr15:89,786,999
Total count: 104
A : 0
C : 101 (97%, 100+, 1-)
G : 3 (3%, 3+, 0-)
T : 0
N : 0
No presence of SNP at CpG site
Patient MET-56. Depth site: 104X. % Meth: 7%

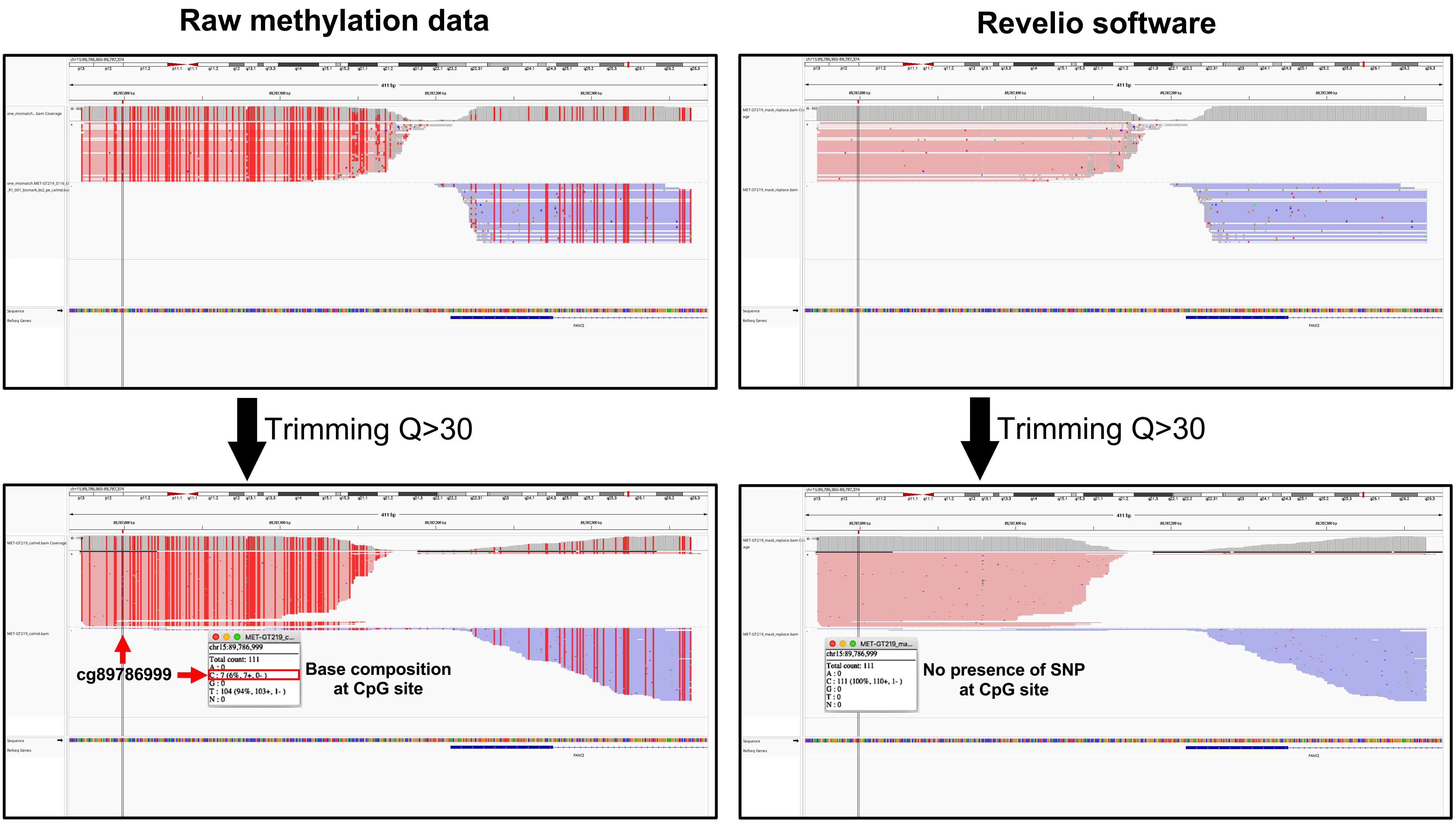

Patient MET-GT219. Depth site: 111X. % Meth: 6.3%

(B) Controls without methylated in cg89786999 site of *FANCI*

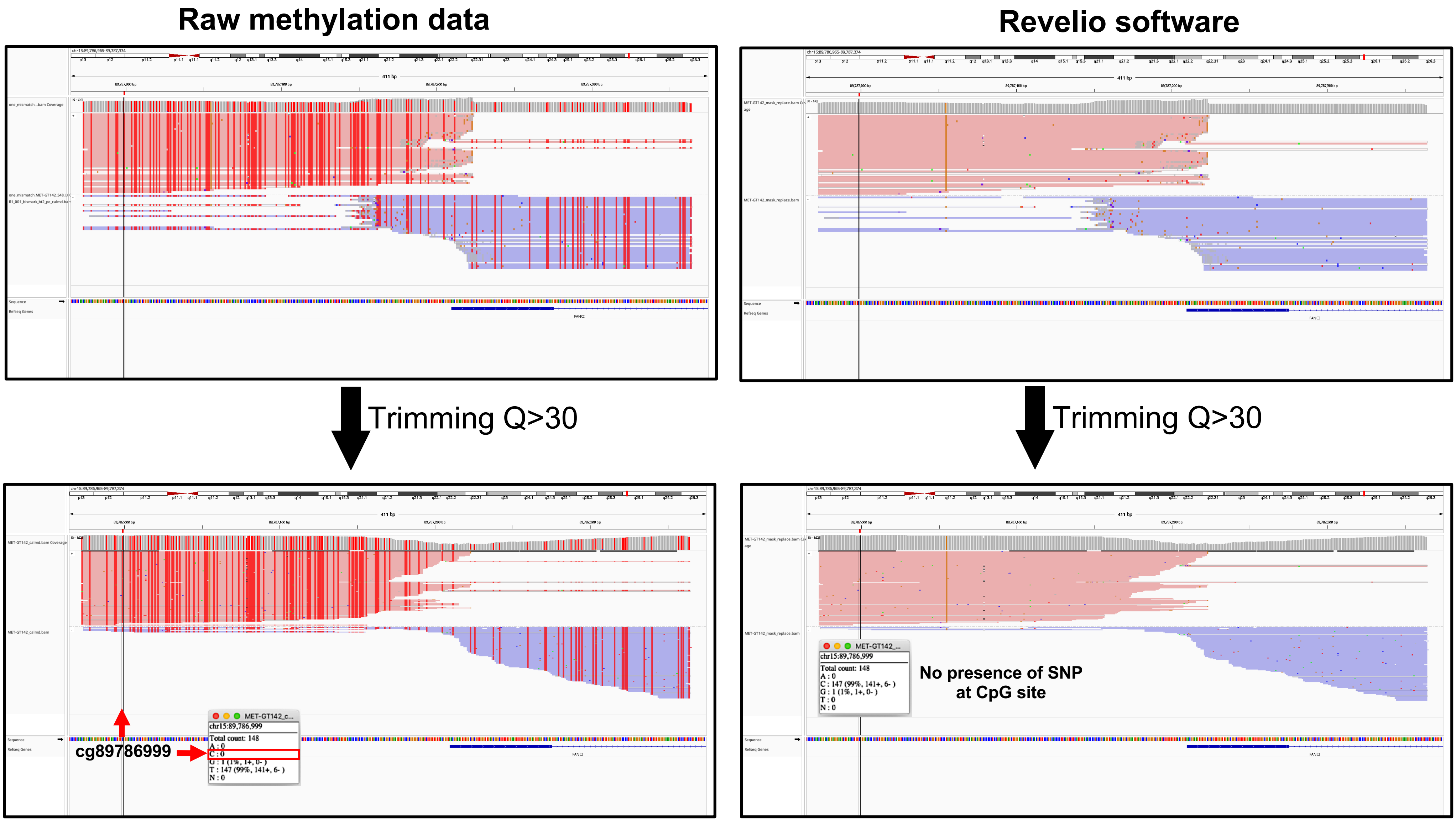

Control MET-GT142. Depth site: 148X. % Meth: 0%

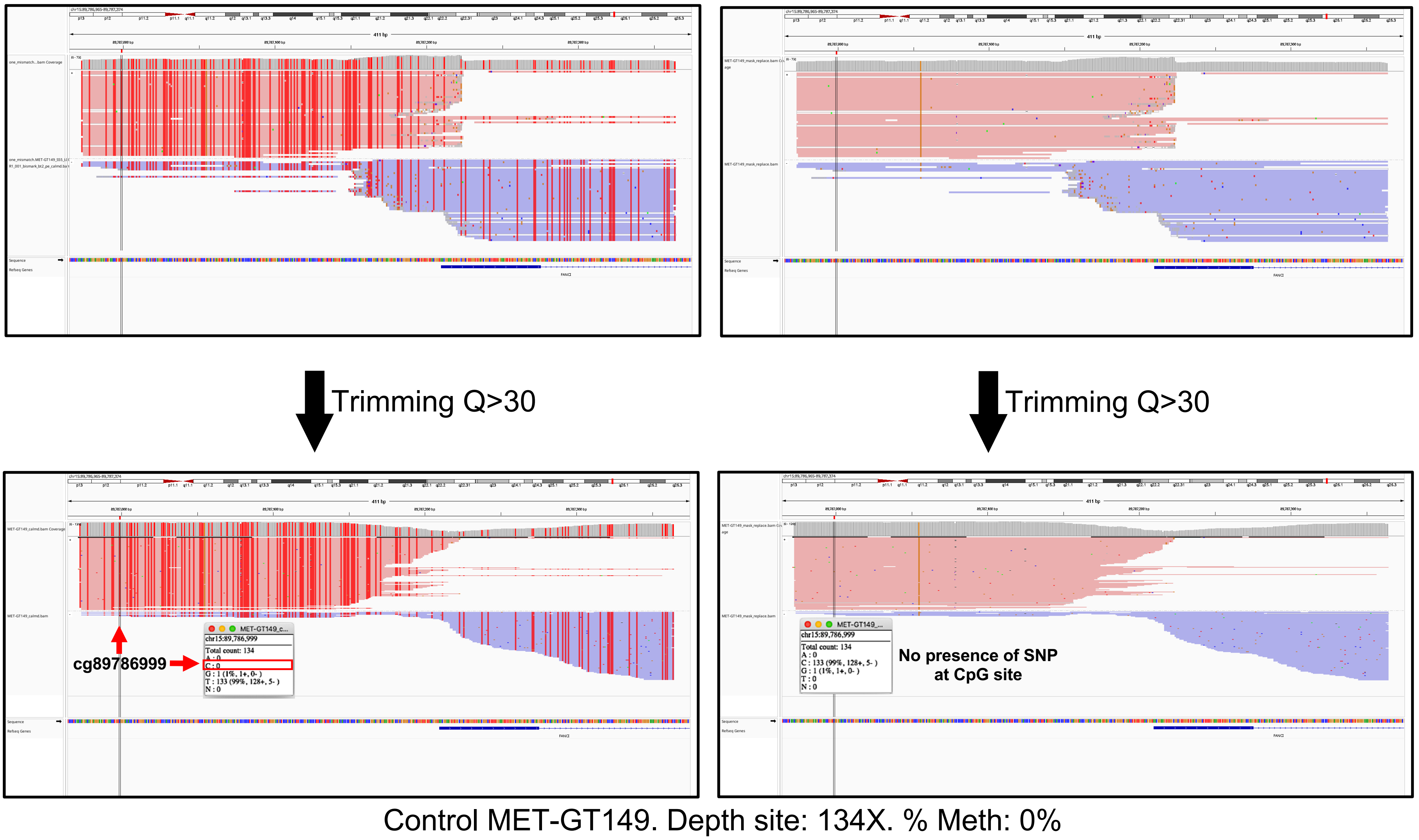

Figure S6. *FANCI* Promoter Region Sequencing. (A) Two representative IGV images are presented for patients with hypermethylation at the cg89786999 site of *FANC*I. **(B)** Two representative IGV images are illustrated for controls without hypermethylation at the site. In the left panel, sequences with raw methylation data (treated with sodium bisulfite) are visualized. The C>T change in the sense strand (*FANCI* gene location with positive polarity) is observed in red due to bisulfite conversion at unmethylated sites. In this case, cytosines resistant to treatment are considered methylated. In the right panel, sequences were processed with the Revelio software, enabling the exclusion of SNP presence at the studied methylation site. Both raw methylation sequences and those analyzed by Revelio were included both before trimming (quality control >30) and after trimming (quality control >30).

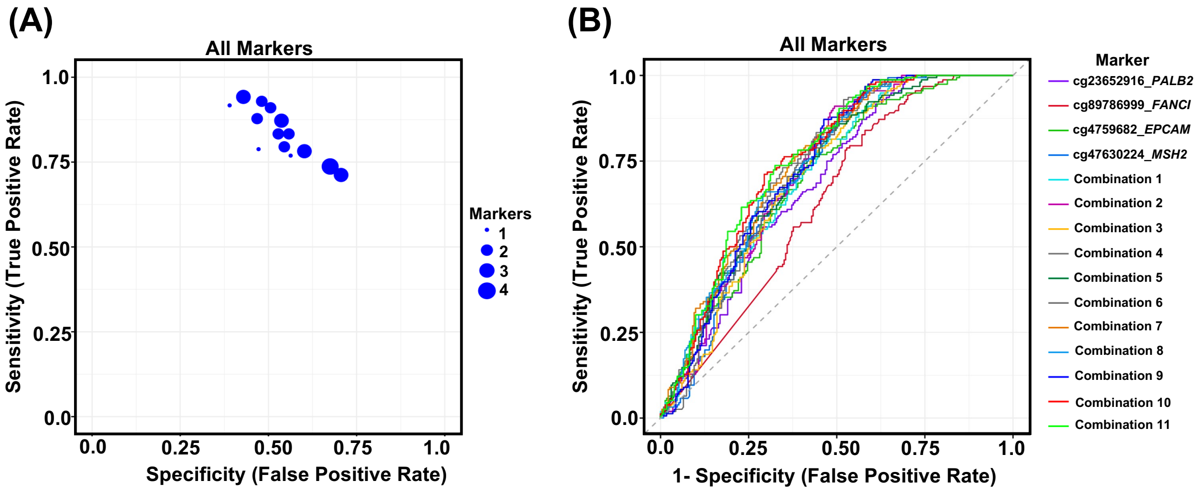

(C)

Marker	Sites CpG in combination	AUC	95% CI	SE	SP	Cut-off
**Combination 1**	cg23652916_PALB2; cg47596828_EPCAM	0.721	0.671 – 0.770	0.795	0.545	0.37
**Combination 2**	cg23652916_PALB2; cg47630224_MSH2	0.725	0.675 – 0.774	0.91	0.506	0.357
**Combination 3**	cg23652916_PALB2; cg89786999_FANCI	0.706	0.656 – 0.757	0.878	0.468	0.336
**Combination 4**	cg47596828_EPCAM; cg47630224_MSH2	0.741	0.693 – 0.789	0.833	0.558	0.38
**Combination 5**	cg47596828_EPCAM; cg89786999_FANCI	0.722	0.672 – 0.771	0.833	0.528	0.339
**Combination 6**	cg47630224_MSH2; cg89786999_FANCI	0.725	0.676 – 0.774	0.929	0.481	0.325
**Combination 7**	cg23652916_PALB2; cg47596828_EPCAM; cg47630224_MSH2	0.746	0.698 – 0.794	0.782	0.602	0.404
**Combination 8**	cg23652916_PALB2; cg47596828_EPCAM; cg89786999_FANCI	0.736	0.687 – 0.784	0.942	0.429	0.285
**Combination 9**	cg23652916_PALB2; cg47630224_MSH2; cg89786999_FANCI	0.732	0.683 – 0.781	0.872	0.537	0.368
**Combination 10**	cg47596828_EPCAM; cg47630224_MSH2; cg89786999_FANCI	0.753	0.706 – 0.801	0.712	0.706	0.447
**Combination 11**	cg23652916_PALB2; cg47596828_EPCAM; cg47630224_MSH2; cg89786999_FANCI	0.758	0.711 – 0.804	0.737	0.675	0.433

Figure S7. Combinatorial analysis of the markers. **A)** Eleven possible combinations were evaluated. The size of the bubbles is proportional to the number of combined markers (one to four). **B)** Biomarker capacity of combined all CpG sites. **C)** ROC curve data from the combinatorial analysis of all sites are shown.
